# Post-stroke cognitive impairment: exploring molecular mechanisms and omics biomarkers for early identification and intervention

**DOI:** 10.3389/fnmol.2024.1375973

**Published:** 2024-05-23

**Authors:** Qiuyi Lu, Anqi Yu, Juncai Pu, Dawei Chen, Yujie Zhong, Dingqun Bai, Lining Yang

**Affiliations:** ^1^Department of Rehabilitation, The First Affiliated Hospital of Chongqing Medical University, Chonging, China; ^2^Department of Neurology, The First Affiliated Hospital of Chongqing Medical University, Chonging, China

**Keywords:** post-stroke cognitive impairment, omics, biomarkers, metabolomics, proteomics

## Abstract

Post-stroke cognitive impairment (PSCI) is a major stroke consequence that has a severe impact on patients’ quality of life and survival rate. For this reason, it is especially crucial to identify and intervene early in high-risk groups during the acute phase of stroke. Currently, there are no reliable and efficient techniques for the early diagnosis, appropriate evaluation, or prognostication of PSCI. Instead, plenty of biomarkers in stroke patients have progressively been linked to cognitive impairment in recent years. High-throughput omics techniques that generate large amounts of data and process it to a high quality have been used to screen and identify biomarkers of PSCI in order to investigate the molecular mechanisms of the disease. These techniques include metabolomics, which explores dynamic changes in the organism, gut microbiomics, which studies host–microbe interactions, genomics, which elucidates deeper disease mechanisms, transcriptomics and proteomics, which describe gene expression and regulation. We looked through electronic databases like PubMed, the Cochrane Library, Embase, Web of Science, and common databases for each omics to find biomarkers that might be connected to the pathophysiology of PSCI. As all, we found 34 studies: 14 in the field of metabolomics, 5 in the field of gut microbiomics, 5 in the field of genomics, 4 in the field of transcriptomics, and 7 in the field of proteomics. We discovered that neuroinflammation, oxidative stress, and atherosclerosis may be the primary causes of PSCI development, and that metabolomics may play a role in the molecular mechanisms of PSCI. In this study, we summarized the existing issues across omics technologies and discuss the latest discoveries of PSCI biomarkers in the context of omics, with the goal of investigating the molecular causes of post-stroke cognitive impairment. We also discuss the potential therapeutic utility of omics platforms for PSCI mechanisms, diagnosis, and intervention in order to promote the area’s advancement towards precision PSCI treatment.

## 1 Introduction

Post-stroke cognitive impairment (PSCI) is defined as a clinical syndrome characterized by cognitive damage that occurs after a stroke and persists for up to 6 months (Mijajlović et al., 2017). Large cohort studies show that between 24 and 53.4% of people have PSCI (Douiri et al., 2013; Lo et al., 2019), yet there are still no accurate billable codes in ICD-11. Compared to stroke survivors without cognitive impairment, individuals with cognitive impairment had significantly greater rates of disability and mortality (Gaynor et al., 2018). PSCI has numerous negative effects on stroke survivors’ functional recovery, including executive function, attention (Aam et al., 2020), spatial ability, language, and executive ability (Srikanth et al., 2003). Furthermore, little is known about the pathophysiology of PSCI, which can be brought on by a number of factors such as cerebral small blood vessel disease (Teng et al., 2017), neuroanatomical lesions (Sun et al., 2014), neuroinflammation, and oxidative stress (Zhang X. et al., 2021). In clinical settings, doctors typically use imaging and scale assessments to diagnose patients (Potter et al., 2021), which the accuracy and objectivity are easily affected by the patient’s education and age (Kim et al., 2022), so it is critical since to find objective biomarkers. On top of that, there is still much to learn about the disease’s molecular mechanisms. A thorough understanding of the molecular mechanisms behind PSCI will facilitate the identification of reliable biomarkers to aid in disease prognosis, therapy, and prevention as well as to diagnose and track the progression of illnesses.

Given the complexity of PSCI’s pathophysiology and the limitations of current diagnostic methods, there is a pressing need to explore innovative approaches. Omics is defined as the probing and analysis of large amounts of data on the entire constitutive structure and function of a given biological system at a specific level (Dai and Shen, 2022). As significant fields of omics, metabolomics, microbiomics, genomics, transcriptomics, and proteomics have made irreversible contributions to the hunt for biomarkers and underlying molecular mechanisms of diseases throughout the past few decades (Fu et al., 2010; Chen and Wu, 2012). Each omics approach delivers biological insights at distinct phases (Bjerrum et al., 2008), enhancing PSCI comprehension. Metabolomics helps identify neurological biomarkers (Zhang et al., 2013) by linking metabolic activity to genetic and environmental variables (Fiehn, 2002).Similarly, microbiomics helps analyze the interactions between microbial communities and the human body, offering insights into neurological dysfunctions (Layeghifard et al., 2017; Sorboni et al., 2022). Genomics and transcriptomics, studying genetic variants and gene expression patterns respectively, have identified key molecular alterations associated with stroke and its cognitive aftermath (Lockhart and Winzeler, 2000; Tan et al., 2021; Li W. et al., 2022; Tsimberidou et al., 2022; Ya et al., 2023). Lastly, proteomics, by mapping protein interactions and functions, supports the diagnosis and development of targeted therapies for neurological disorder (Rohlff, 2001; Sancesario and Bernardini, 2018). Although omics approaches are crucial for deepening our understanding of PSCI, the proliferation of omics research in neurological disorders is impeded by technical difficulties, limited clinical applicability, and a lack of comprehensive reviews specifically focusing on post-stroke cognitive impairment.

This study aimed to identify multi-omics alterations in patients with PSCI. In order to assist in characterizing possible biomarkers with prospective applications such as early diagnosis and tracking disease progression, we offer an overview of the multi-omics attributed with post-stroke cognitive impairment. Additionally, the obstacles inherent in biomarker research in PSCI are brought to light so that we can work towards advancing precision medicine in PSCI. We also illustrate the therapeutic relevance of the omics platform for the pathogenesis, diagnosis, and treatment of PSCI, as a means to establish the groundwork for an extensive investigation of PSCI.

## 2 Materials and methods

### 2.1 Diagnostic biomarker

All published articles from database inception to October 2023 were searched using our listed term combinations (Supplementary Tables S1–S5) in electronic databases such as PubMed, Cochrane Library, Embase, Web of Science, and common databases for each omics. We then used a snowballing strategy to expand the search. We adopted standard inclusion criteria for the selection of studies: (1) all tissue types belonging to PSCI patients; (2) differential biomarker detection using omics techniques; (3) clinical trial studies assessing changes in levels of various types of biomarkers in patients with PSCI; and (4) peer-reviewed full-text papers published in English. These were the conditions for exclusion: (1) interventional studies; (2) non-human studies; (3) non-original research (reviews, case reports, etc.). Two authors (QL and AY) independently performed screening based on inclusion and exclusion criteria. All disagreements experienced were resolved through ongoing discussions with all authors.

### 2.2 Data extraction and processing

Excel software was implemented to manually organize the data and perform statistical analysis (Microsoft, Ver. 2019). We separately retrieved data from publications that qualified using a pre-designed table. Elected information consisted of three sections: (1) article information including title, first author, year of publication, and region of recruitment; (2) patient information including sample size, sex ratio, mean age, stroke type, and cognitive function assessment tool; and (3) biomarker information included sample type and selected metabolites that were statistically significant (*p* < 0.05 was considered statistically significant). The pictures for this article were drawn in Adobe Photoshop and Adobe Illustrator.

## 3 Results

### 3.1 Literature search results

**Figure 1:**
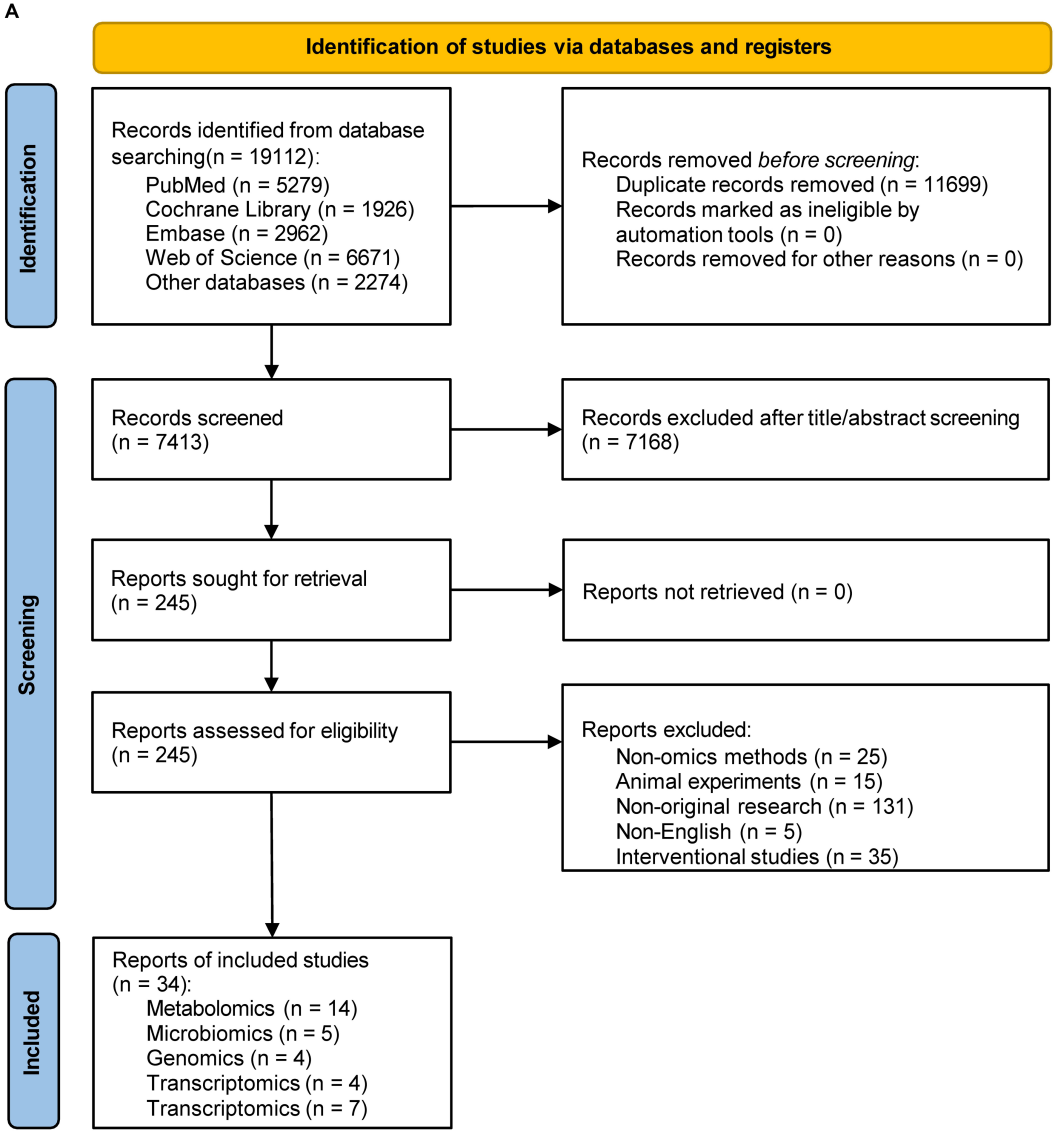

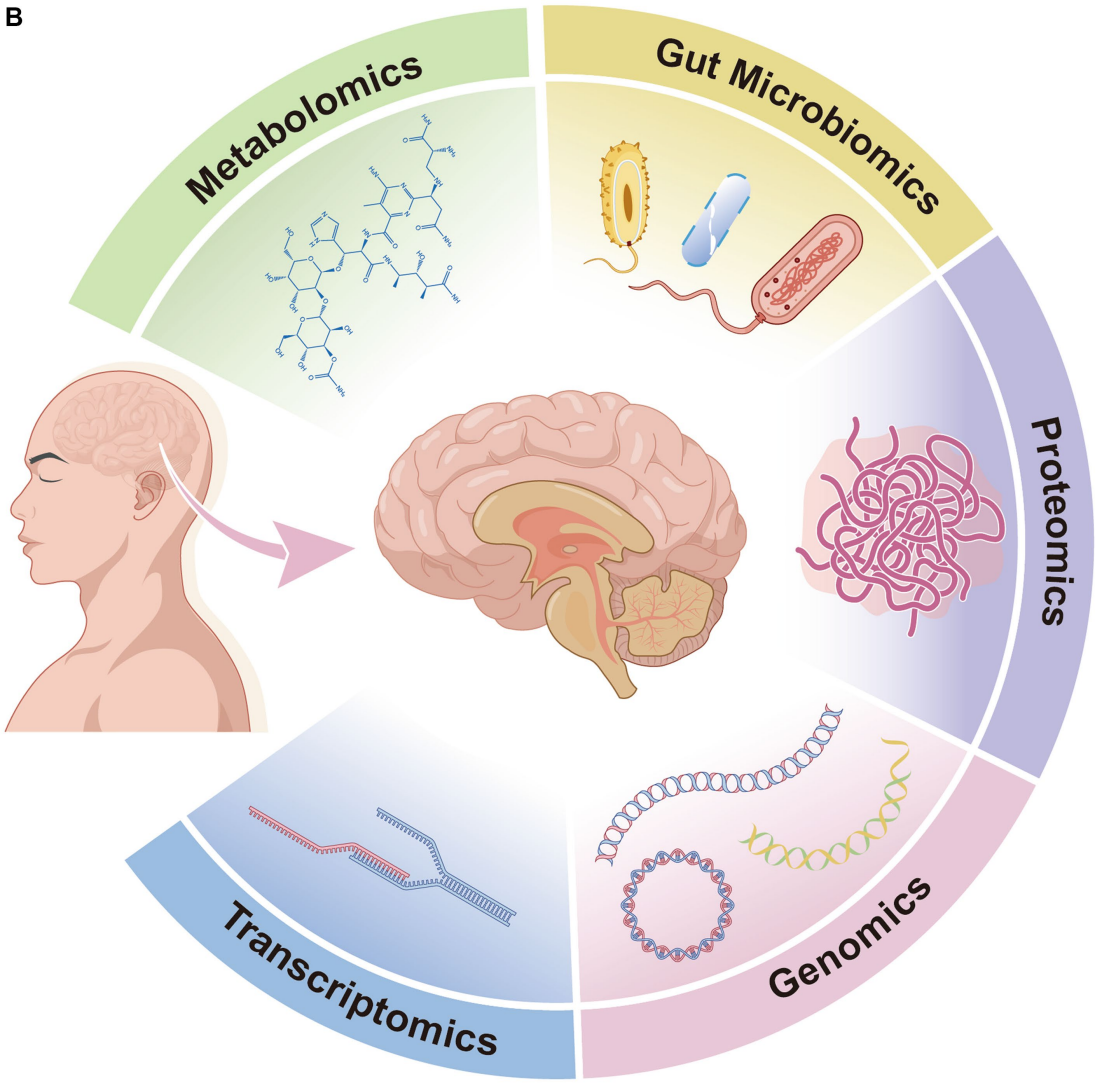
**(A)** Flow diagram of literature search and study selection. **(B)** Overview of multi-omics of post-stroke cognitive impairment.

We systematically retrieved a total of 19,112 records in PubMed, Cochrane Library, Embase, and Web of Science databases (Figure 1A; the outcomes of each omics search are shown in Supplementary Tables S6–S10). After a first screening of titles and abstracts, a second assessment of the entire text, and additional resources, 34 papers were retained based on our predetermined inclusion and exclusion criteria. Out of the results we kept, 14 publications described metabolomics (one of which also discussed microbiomics), 5 articles covered microbiomics, 4 papers concerned genomics, 4 articles concerned transcriptomics, and 7 studies described proteomics (Figure 1B).

### 3.2 Characteristics of the included studies

Ultimately, 34 articles were included in our study, with publication years between 2011 and 2023. The features of the included studies are displayed in Table 1. Of these articles meeting the criteria, 23 (67.65%) were undertaken in China, the rest in Poland (2, 5.88%), the United States (2, 5.88%), France, Switzerland, Canada, Iran, Korea, Indonesia, Singapore, and other countries. We only counted the sample size once in the following research since two Polish studies that analyzed the same sample of subjects produced different results. A total of 4,223 stroke patients and 702 healthy controls were involved in the 34 included studies, with sample size ranging from 10 to 617. Of these, the patient source for 26 research was solely ischemic stroke, 1 study was hemorrhagic stroke, and 7 studies were not defined. Subjects were assessed neuropsychologically primarily using the Montreal Cognitive Assessment (MoCA) scale or Mini-Mental State Examination (MMSE) scale, but the study from Poland also used the California Verbal Learning Test 2nd (CVLT2) to assess specific abilities.

**Table 1:** Basic information for omics studies.

Post-stroke cognitive impairment		Non-post-stroke cognitive impairment		Healthy control		Issue type	Patience source	Stroke type	Reference
Sample size (F/M)	Average age (year)^a^	Sample size (F/M)	Average age (year)^a^	Sample size (F/M)	Average age (year)^a^				
72 (40/32)	60.70	NA	NA	30 (18/12)	63.1	Serum	Poland	IS	Kotlęga et al. (2021b)
617 (184/433)	60.0 ± 10.5	NA	NA	NA	NA	Plasma	China	IS	Che et al. (2022)
73 (40/33)	63.40	NA	NA	30 (18/12)	63.1	Plasma	Poland	IS	Kotlęga et al. (2021a)
86 (36/50)	71.10 ± 10.40	170 (81/89)	65.00 ± 10.80	100 (NA)	NA	Plasma	China	IS	Zhu et al. (2020)
20 (10/10)	66.10 ± 6.50	20 (10/10)	67.50 ± 8.64	20 (10/10)	67.30 ± 6.81	Serum	China	IS	Liu et al. (2015)
30 (5/25)	64.90 ± 8.13	35 (11/24)	64.06 ± 8.67	NA	NA	Fecal	China	IS	Liu et al. (2020a)
617 (184/433)	60.00 ± 10.50	NA	NA	NA	NA	Plasma	China	IS	Zhong et al. (2021)
99 (46/53)	65.30 ± 9.03	83 (24/59)	61.27 ± 10.17	NA	NA	Serum	China	IS	Feng et al. (2020)
122 (42/80)	64.32 ± 9.82	106 (25/81)	60.55 ± 11.03	NA	NA	Plasma	China	IS	Gong et al. (2021)
40 (21/19)	61.32	NA	NA	20 (9/11)	61.03	Serum	China	IS	Wang X. et al. (2022)
13 (5/8)	69.40 ± 17.80	10 (4/6)	64.70 ± 13.30	NA	NA	Serum	France	IS	Cogo et al. (2021)
149 (97/52)	81.04 ± 5.30	NA	NA	NA	NA	Serum	Sweden	IS	Pascoe and Linden (2016)
36 (16/20)	59.00 ± 9.3	36 (17/19)	59.300 ± 8.20	NA	NA	Brain	China	IS	Meng et al. (2016)
41 (19/22)	72.30 ± 12.20	NA	NA	NA	NA	Plasma	Canada	IS	Gold et al. (2011)
34 (24/10)	61.50	49 (44/5)	54.00	NA	NA	Fecal	China	IS, HS	Wang H. et al. (2022)
53 (18/35)	72.20 ± 10.30	40 (13/27)	66.00 ± 10.80	NA	NA	Fecal	China	IS	Ling et al. (2020a)
41 (24/17)	69.63 ± 9.35	25 (11/14)	68.92 ± 8.46	NA	NA	Fecal	China	IS	Ling et al. (2020b)
29 (9/20)	NA	27 (13/14)	NA	19	NA	Fecal	China	IS	Huang et al. (2021)
86 (36/50)	63.12 ± 12.47	NA	NA	NA	NA	Blood	America	IS, HS	Han Z. et al. (2020)
48 (34/14)	74.52 ± 8.80	158 (93/65)	61.49 ± 10.78	NA	NA	Blood	Iran	IS, HS	Rezaei et al. (2016)
81 (20/61)	71.40 ± 11.32	NA	NA	NA	NA	Serum	China	IS	Zeng et al. (2019)
361 (123/238)	63.80 ± 9.60	NA	NA	346 (155/191)	60.6 ± 10.6	Blood	China	IS	Han Y. et al. (2020)
36 (20/16)	68.00	38 (23/15)	67.00	NA	NA	Plasma	China	IS, HS	Wang et al. (2020)
45 (NA)	NA	32	NA	NA	NA	Serum	China	IS, HS	Yuan et al. (2022)
108 (41/67)	53.78 ± 11.32	NA	NA	72 (37/39)	54.07 ± 11.18	Blood	China	IS, HS	Zhai et al. (2020)
39 (19/20)	66.20 ± 8.80	37 (17/20)	65.80 ± 9.20	38 (18/20)	65.6 ± 7.4	Serum	China	IS, HS	Huang et al. (2016)
35 (11/24)	61.63 ± 8.47	21 (10/11)	58.67 ± 9.01	NA	NA	Serum	Indonesia	IS	Prodjohardjono et al. (2020)
23 (NA)	NA	NA	NA	17 (13/4)	56	Plasma	Singapore	IS	Datta et al. (2022)
61 (NA)	NA				NA	Plasma	America	IS	Hazelwood et al. (2022)
80 (39/41)	64.50 ± 10.20	118 (44/74)	67.60 ± 9.10	NA	NA	Serum	China	IS	Zhang Y. et al. (2021)
192 (72/120)	65.70 ± 7.80	124 (49/75)	65.30 ± 8.20	NA	NA	Serum	China	HS	Gao et al. (2022)
37 (NA)	NA	43 (NA)	NA	NA	NA	Serum	China	IS	Li Z. et al. (2022)
286 (117/189)	NA	NA	NA	NA	NA	Blood	Korea	IS	Kim et al. (2012)
10 (5/5)	60.30 ± 9.17	NA	NA	10 (4/6)	55.80 ± 6.92	Plasma	China	IS	Qi et al. (2023)

a Continuous variables are presented as mean ± SD; NA means not available.

### 3.3 Metabolomics in PSCI

Metabolomics demonstrates the qualitative and quantitative analysis of the dynamic metabolic responses of the human body in response to multifactorial stimuli (Nicholson et al., 1999). Researchers typically isolate small molecules using gas chromatography (Xiayan and Legido-Quigley, 2008), liquid chromatography, or capillary electrophoresis and quantify potential biomarkers using nuclear magnetic resonance spectroscopy or mass spectrometry.

Of the research that we looked into, 14 publications described differential metabolites, with serum (50.0%) and plasma (41.7%) accounting for the majority of biological samples and one (8.3%) for feces. We extracted 54 metabolites from these studies (shown in Table 2), and 51 metabolites in total were selected once duplicates were eliminated.

**Table 2:** Metabolites in PSCI.

Metabolite	Tissue type	Expression	Reference
Arachidonic acid	Serum	Down	Kotlęga et al. (2021b)
Eicosapentaenoic acid	Serum	Down	
Alpha-linolenic acid	Serum	Up	
Stearidonic acid	Serum	Up	
Tricosanoic acid	Serum	Up	
Pentadecanoid acid	Serum	Down	
Gamma-linolenic acid	Serum	Down	
Myristic acid	Serum	Up	
Myristoleic acid	Serum	Up	
Vaccenic acid	Serum	Up	
Arachidic acid	Serum	Up	
L-carnitine^a^	Plasma	Down	Che et al. (2022)
Prostaglandin E2	Plasma	Up	Kotlęga et al. (2021a)
9-hydroxyoctadecadienoic acid	Plasma	Up	
13-hydroxyoctadecadienoic acid	Plasma	Up	
5-hydroxyeicosatetraenoic acid	Plasma	Up	
12-hydroxyeicosatetraenoic acid	Plasma	Up	
Maresin 1	Plasma	Up	
Leukotriene B4	Plasma	Up	
Resolvin D1	Plasma	Down	
Trimethylamine N-oxide^a^	Plasma	Up	Zhu et al. (2020)
L-carnitine^a^	Serum	Up	Liu et al. (2015)
Creatine	Serum	Up	
L-glutamine	Serum	Up	
L-proline	Serum	Up	
N-acetylneuraminic acid	Serum	Up	
Hypoxanthine	Serum	Up	
Uric acid	Serum	Up	
L-tyrosine	Serum	Up	
L-kynurenine^a^	Serum	Up	
L-phenylalanine	Serum	Up	
Sphingosine-1-phosphate	Serum	Up	
L-palmitoylcarnitine	Serum	Up	
Citric acid	Serum	Down	
L-valine	Serum	Down	
L-isoleucine	Serum	Down	
L-tryptophan	Serum	Down	
LysoPCs	Serum	Down	
Stearoylcarnitine	Serum	Down	
Acetic acid	Plasma	Up	Liu et al. (2020a)
Acetic acid	Fecal	Down	
Propionic acid	Fecal	Down	
Isobutyric acid	Fecal	Down	
Butyric acid	Fecal	Down	
Isovaleric acid	Fecal	Down	
Valeric acid	Fecal	Down	
Caproic acid	Fecal	Down	
Choline	Plasma	Down	Zhong et al. (2021)
Betaine	Plasma	Down	
Thiamine	Serum	Down	Feng et al. (2020)
Trimethylamine N-oxide^a^	Plasma	Up	Gong et al. (2021)
L-glutamate	Serum	Up	Wang X. et al. (2022)
L-kynurenine^a^	Serum	Up	
Quinolinic acid	Serum	Up	Cogo et al. (2021)
Methylmalonic acid	Serum	Up	Pascoe and Linden (2016)

a For the duplicate metabolites.

There is now more evidence linking B vitamins to PSCI. For example, in patients with cerebral infarction, thiamine deficiency is predictive with early cognitive impairment (Feng et al., 2020). It has also been demonstrated that folic acid, together with its serum-specific marker methylmalonic acid (MMA), can predict (Pascoe and Linden, 2016) and help diagnose (Durga et al., 2007) cognitive function in patients with post-stroke. Following a stroke, it was discovered that folic acid metabolism products, choline and betaine (Ueland, 2011), had a negative correlation with cognitive performance (Zhong et al., 2021).

One of the possible pathologic mechanisms of PSCI is the generation of inflammation after stroke (Zhang X. et al., 2021), which activates indoleamine 2,3-dioxygenase (IDO), whose activity is associated with cognitive impairment (Gold et al., 2011; Cogo et al., 2021). IDO leads to increased production of kynurenine, while kynurenine (Sapko et al., 2006) and quinolinic acid (Santamaría et al., 2001; Stone and Darlington, 2002) are involved in the induction of synaptic plasticity, and changes in these two substances’ concentrations and ratios have been considered accurate indicators of PSCI (Cogo et al., 2021). Serum glutamate levels in PSCI patients further support the idea (Wang X. et al., 2022) that although glutamate is an excitatory neurotransmitter linked to cognitive function, excessive quantities might cause severe excitotoxicity that can aggravate ROS and inflammation (Liu et al., 2020b). Meng et al. (2016) found that PSCI patients had a considerably lower N-acetylaspartic acid/creatine ratio in their hippocampal regions, whereas Liu et al. (2015) also discovered an increase in serum creatine. Several other alterations in amino acid levels have likewise been suggested to be associated with PSCI (Liu et al., 2015), including glutamine, proline, tyrosine, phenylalanine, isoleucine, tryptophan, valine, and N-acetylneuraminic acid. Notably, PSCI was negatively correlated with L-carnitine (Liu et al., 2015; Che et al., 2022), which is produced naturally from two necessary amino acids (Tein et al., 1993). This is probably because L-carnitine has a greater ability to cross the blood–brain barrier and shields neuronal cells from ischemia injury (Zhang R. et al., 2012; Ribas et al., 2014).

PSCI pathophysiology involves the metabolic cascade of polyunsaturated fatty acids (Baierle et al., 2014). Whereas alpha-linolenic acid acts as a substrate for gamma-linolenic acid, which then produces arachidonic acid through a sequence of reactions (Kotlega et al., 2020), it has been demonstrated that this inflammatory cascade is linked to cognitive impairment in stroke survivors (Kotlęga et al., 2021b). Arachidonic acid may also serve as a bridge between lipid metabolism, neuroinflammation, and cognitive function in the pathophysiology of ischemic stroke (Szczuko et al., 2020). Prostaglandins formed from arachidonic acid are key mediators of neuroinflammation, among which prostaglandin E2, 9S-HODE, and 13S-HODE were found to be significantly correlated with PSCI (Kotlęga et al., 2021a).

Apart from the metabolites present in blood samples of patients, the metabolomics of PSCI are also influenced by differential metabolites that are broken down by gut microbes. These metabolites include butyrate (Wang H. et al., 2022), lipopolysaccharide, trimethylamine-n-oxide (TMAO) (Zhu et al., 2020; Gong et al., 2021), and seven major short-chain fatty acids (Liu et al., 2020a), which are acetic acid, propionic acid, isobutyric acid, butyric acid, isovaleric acid, valeric acid, and caproic acid.

Heterogeneity in current metabolomics research on PSCI is challenging to avoid because of variations in areas, sample sizes, and analytical techniques. Secondly, most of the selected studies are blood studies, and there are no metabolomics studies of brain tissue and other organs yet. Thirdly, there is a wide variety of metabolites and a lack of replicability. Fourthly, there aren’t many viable biomarkers for PSCI research in nuclear magnetic resonance spectroscopy, one of the potent metabolomics techniques. We believe that future research directions for study could focus on enhancing these elements and delving deeper into PSCI therapy that targets lipid level alterations.

### 3.4 Gut microbiomics in PSCI

Microbiomics is an emerging field of research aimed at identifying the components of the microbiome, characterizing the interactions between the microbiome and the host, and determining its impact on disease (Barko et al., 2018). The most used techniques are bacterial 16s ribosomal RNA genome sequencing (Wang and Qian, 2009) and shotgun sequencing (Quince et al., 2017).

Among the five studies on gut microbiomics that were considered, we found divergent microbiota in two phyla, one class, two orders, three families, and six genera. The emergence of the gut-brain axis (GBA) has enhanced our understanding of neurological disease progression. The gut microbiota may interact with GBA through autonomic, endocrine, and immune crosstalk (Zhao et al., 2021). Therefore, researchers also paid attention to the gut microbiome of PSCI patients. *Bacillota* (Liu et al., 2020a), *Proteobacteria* (Ling et al., 2020a,b) and *Bacteroidete*s (Liu et al., 2020a) at the phylum level in PSCI patients. *Gammaproteobacteria* was negatively correlated with MoCA scores in patients with post-stroke comorbid cognitive impairment and depression (Ling et al., 2020a,b). Patients with PSCI exhibited notable visual changes in *Enterobacterales* and *Lactobacillales*, which were linked to inflammation (Ling et al., 2020a). Whereas the abundance of *Enterobacteriaceae* (Huang et al., 2021; Wang H. et al., 2022), *Streptococcaceae*, and *Lactobacillaceae* increased significantly in PSCI patients (Ling et al., 2020a). At the genus level, *Fusobacterium* (Liu et al., 2020a), *Streptococcus*, *Klebsiella*, *Lactobacillus* (Ling et al., 2020a) and *Enterococcus*, *Bacteroides* (Huang et al., 2021) were significantly altered in post-stroke patients. Patients with PSCI exhibited a specific deficiency in microorganisms that produce short-chain fatty acids (SCFAs), including *Oscillibacter*, *Ruminococcus*, *Gemmiger*, *Coprococcus*, and *Barnesiella* (Liu et al., 2020a). SCFAs can cross the blood-brain barrier into the brain and act in the central nervous system (Kekuda et al., 2013), which also confirms the gut-brain axis of bidirectional communication.

The results of changed biodiversity in the PSCI microbiome varied, depending on a number of variables including age, antibiotic use, and diet (Lozupone et al., 2012). The present study did not exclude the influence of the aforementioned factors, and the cross-sectional study design made it impossible to establish a causal link between PSCI and changes in the microbiota. We further propose that, upon the present flaws have been addressed, future research should concentrate on delving deeper into the connections between PSCI and the gut microbiota in combination with other omics analyses.

### 3.5 Genomics in PSCI

High-throughput sequencing technology has made human diseases easier to investigate in recent years by giving researchers a deeper and more comprehensive platform to examine disease processes and underlying mechanisms. Genetic linkage analysis, candidate gene studies, genome-wide association studies (GWAS), and next-generation sequencing techniques (NGS) have become the main means to study disease etiology and risk genes (Giri et al., 2016).

Out of the 5 genomics studies we found, 4 genes were described to be significantly associated with PSCI. Brain-derived neurotrophic factor (BDNF) promotes neurology and angiogenesis (Kurozumi et al., 2004; Schäbitz et al., 2007) and influences post-stroke recovery through its neuroplastic effects (Lu, 2003). BDNF Val is considered a risk allele for patients with poststroke dementia, and it was found that ischemic stroke patients with a heterozygous Val/Met genotype developed cognitive impairment earlier than those with a Met/Met homozygous genotype and had significantly reduced survival (Kim et al., 2012; Rezaei et al., 2016). The BDNF val66Met single nucleotide polymorphism (SNP) leads to intracellular packaging of proBNDF and secretion of mature BDNF (Chen et al., 2004; Yoshida et al., 2012) and is considered a key predictor of cognitive outcome and functional recovery after stroke in hospitalized patients (Rezaei et al., 2016; Han Z. et al., 2020). The reason may be that BNDF val66Met is associated with the prefrontal cortex, anterior cingulate cortex, and hippocampus volume (Niendam et al., 2012; Balconi, 2013), affecting the neural circuits that control cognition (Schweiger et al., 2019). Serum cystatin C is similarly thought to prevent cognitive impairment by inhibiting amyloid Aβ (Sastre et al., 2004; Kaeser et al., 2007), a protein encoded by the CST3 gene. Zeng et al. (2019) claimed that the CST3B allele or CST3 gene polymorphism could be one of the early diagnostic indicators of PSCI. In addition, apolipoprotein E (APOE), one of the major members of very low-density lipoproteins (Levy et al., 2001), was found to have three alleles (ε2, ε3, and ε4), and APOE ε4 carriers and ε4 alleles are more susceptible to PSCI than healthy populations (Han Y. et al., 2020). APOE ε4 allele has also been suggested to be a biologically active factor for β-amyloid peptide deposition in the brain, which ultimately leads to the narrowing of blood vessels and altered cerebral perfusion (Gurol et al., 2006; Godin et al., 2009).

There are currently no genome-wide investigations of PSCI, and research is restricted to potential genes at this time. Genome-wide data from PSCI patients must be gathered and processed immediately in order to identify all loci of variations linked with PSCI risk and any potential regulatory mechanisms. Finding all degrees of association responses and PSCI mechanisms will be made easier by connecting genetic variations to the development of the disease. In addition, we advocate further exploration of the clinical and pharmacological applications of BDNF for stroke and PSCI, which we believe is a powerful way forward.

### 3.6 Transcriptomics in PSCI

Not only is transcription the initial stage of gene expression, but it is also a crucial regulatory stage. The post-genome age has seen a rise in interest in transcriptomics. These days, techniques for analyzing gene expression include RNA sequencing, microarray screening, and real-time PCR (Bagyinszky et al., 2020). Not only that, for microRNA detection, other methods based on nucleic acid amplification have been developed (Ye et al., 2019), including rolling loop amplification (RLA), double-stranded specific nuclease (DSN)-based amplification, loop-mediated isothermal amplification (LAMP), etc.

Six distinct miRNAs were proposed as PSCI biomarkers from a total of 4 transcription-related studies that were deemed relevant to patients with PSCI. MicroRNA (miRNA) is increasingly being recognized as a novel biomarker and therapeutic target for a variety of diseases, including ischemic stroke (Yang S. et al., 2020). MiRNA dysregulation provides early warning signals of brain disease outwardly by causing changes in mRNA through exosomes in concert with proteins (Zhang et al., 2015). MiRNA-132 interferes with neuronal maturation by affecting dendritic arborization and spinogenesis and has been demonstrated to exist as a key activity-dependent regulator of cognition (Hansen et al., 2013). The predictive effect of miRNA-132 on PSCI has been found (Huang et al., 2016; Yuan et al., 2022). Clinical studies have shown that miRNA-21 attenuates brain injury and neurological dysfunction through neurogenesis and angiogenesis (Lopez et al., 2017), and its upregulation is significantly associated with an increased risk of PSCI (Yuan et al., 2022). Moreover, miRNA-200b was found to be associated with the vascular endothelial growth factor A gene (Li et al., 2017), which can enhance vascular permeability, and its expression level was also found to be up-regulated in PSCI patients (Yuan et al., 2022). Also capable of regulating VEGF expression was miRNA-195 (Wang et al., 2013), which was also found to be associated with PSCI by Zhai et al. (2020). There are also miRNAs such as miRNA-497 (Zhai et al., 2020) and miRNA-let-7i (Wang et al., 2020) that were found to be significantly associated with post-stroke cognitive function in PSCI patients.

Both non-protein-coding and protein-coding RNA are included in the entire gene expression profile linked to PSCI. Unfortunately, the present study was limited to miRNA. The supremacy of transcriptome development must be understood in order to facilitate the deciphering of PSCI molecular networks. To provide a more thorough understanding, we propose that future efforts be directed towards investigating the entire range of gene expression in PSCI.

### 3.7 Proteomics in PSCI

Proteomics is the characterization of the proteome, including the expression, structure, function, interactions, and modifications of proteins at any stage (Domon and Aebersold, 2006). Proteomics plays a key role in early diagnosis, prevention, and tracking of the progress of diseases. Proteomics assay techniques are complex and numerous, such as chromatography-based purification, electrophoresis-based separation, and high-throughput technologies.

Seven research on PSCI proteomics have been reported to date. One of the main roles that inflammation appears to have in the pathophysiology of PSCI. Serum amyloid A is involved in the chemotactic recruitment of inflammatory cells (Sack, 2018) and has been shown to play a role in various central nervous system diseases (Kisilevsky and Manley, 2012), including PSCI (Zhang Y. et al., 2021). The upregulation of the inflammatory protein GP1BA, which binds to the von Willebrand factor and is involved in platelet adhesion and activation (Kroll et al., 1991), indicates a pro-inflammatory response of the immune system to ischemic injury and promotes the occurrence of PSCI (Hazelwood et al., 2022). The proteins ARTN, HGF (Gallo et al., 2015), and VEGF (Lee et al., 2010) are associated with angiogenesis and neuroprotection, and their early predictive role in PSCI patients was identified (Prodjohardjono et al., 2020; Hazelwood et al., 2022). Both neuroglobin and hemoglobin, which are subtypes of pearl proteins (Lopez et al., 2010; Baez et al., 2016), play an important role in oxidative stress and protect brain tissue from hypoxic and ischemic damage, so their reduced levels increase the risk of developing PSCI (Gao et al., 2022; Li Z. et al., 2022).

According to Qi et al. (2023), 31 proteins that were up-regulated in PSCI patients compared to controls may be connected to platelet aggregation and coagulation, whereas 128 proteins that were down-regulated may be connected to pathways such complement activation, fibrin clotting, and cell adhesion protein binding. However, a quantitative proteomics study of plasma in patients with lacunar infarction identified 112 proteins associated with cognitive decline (Datta et al., 2022), most of which were not associated with inflammation, complement activation, coagulation, fibrinolysis, or endothelium. We hypothesize that this variability exists because of geographic differences, sample selection time, and subject individualization differences.

Based on current research findings, proteins are powerful biomarkers for the diagnosis and prognosis of PSCI. However, the majority of research conducted thus far has utilised blood samples, with only a small number examining additional samples such urine, saliva, brain tissue, and cerebrospinal fluid. We look forward to improving the differential expression analysis and co-expression network analysis of different samples of PSCI patients in the future to further explore the mechanism behind PSCI.

## 4 The clinical application prospect of multi-omics in PSCI

**Figure 2:**
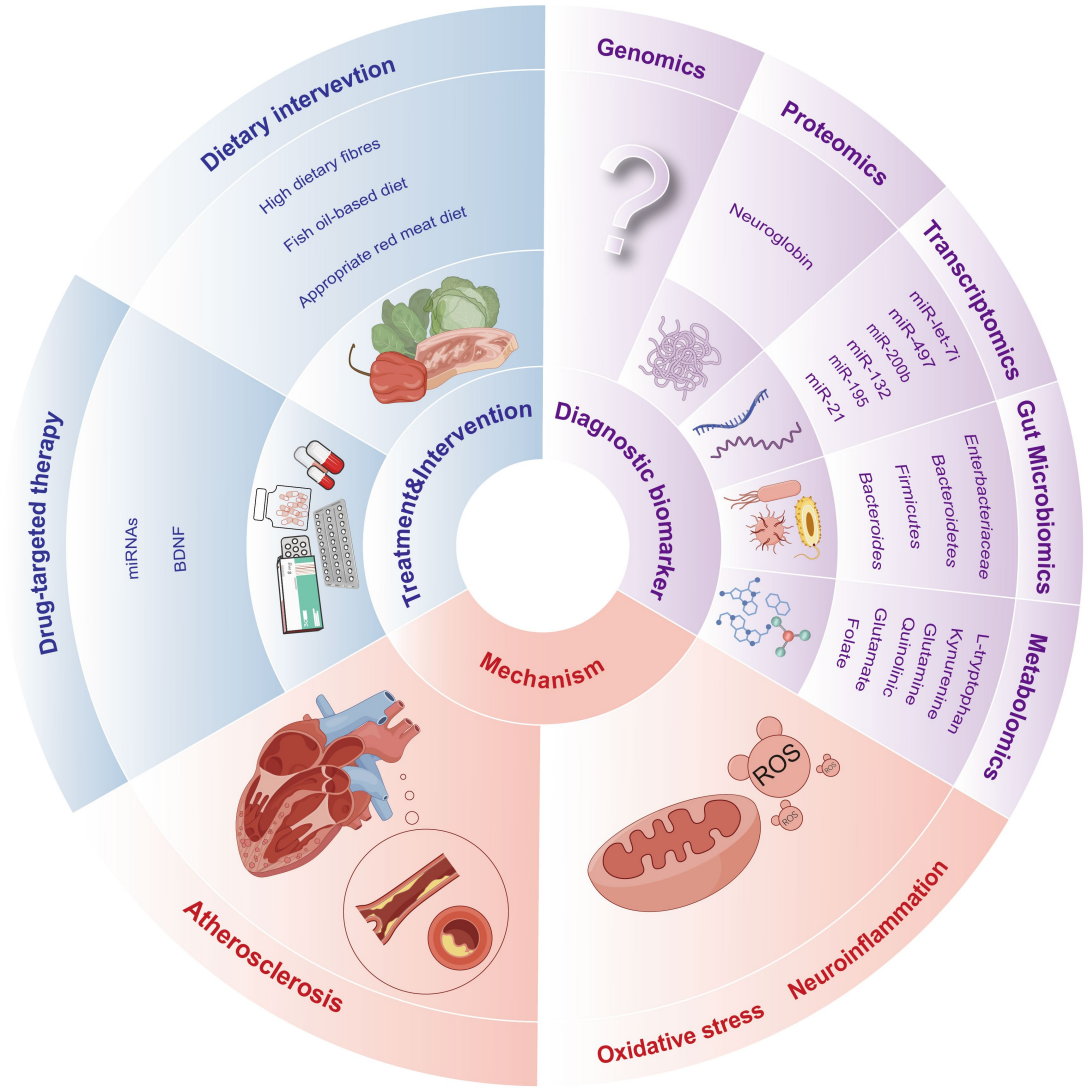
Prospects for clinical applications of the multi-omics of PSCI.

A variety of omics platforms have been developed to detect biomarkers at different levels, enabling us to delve into previously uncharted territories of understanding, such as diagnosis, mechanism exploration and targeted therapy for PSCI (Figure 2).

### 4.1 Potential diagnostic biomarker

Based on the current metabolomics of PSCI, amino acids such as glutamine, kynurenine, and its metabolite quinolinic acid may be employed as PSCI diagnostic indicators. Glutamate has been implicated in the pathophysiology of cerebral ischemia in earlier research (Dirnagl et al., 1999), and it has also been suggested that glutamate may be a biomarker for stroke (Castellanos et al., 2008), Parkinson’s disease (Vascellari et al., 2020), Huntington’s disease, and other neurodegenerative diseases that cause early cognitive dysfunction (Unschuld et al., 2012) due to the excitotoxicity of glutamate (Estrada Sánchez et al., 2008). One of the necessary amino acids, L-tryptophan, can be metabolized to quinolinic acid and kynurenine via the kynurenine pathway. It has been discovered that these metabolites have the ability to distinguish between atherosclerotic and cardiac cerebral infarcts (Lee et al., 2023). Moreover, a number of processes by which the kynurenine pathway causes neurotoxicity and neuronal death (Guillemin, 2012) can contribute to cognitive impairments (Heisler and O'Connor, 2015). Also, folate has been proposed for the diagnosis of PSCI and has previously been found to have potential as a biomarker for ischemic stroke (Sidorov et al., 2019).

Gut microbiomics at PSCI identified microbiota that could equally serve as non-invasive diagnostic biomarkers, including *Firmicutes*, *Bacteroidetes*, *Enterobacteriaceae*, and *Bacteroides*. Current ideas suggest that dysregulation of *Firmicutes/Bacteroidetes* ratio can be used as a potential biomarker of cognitive impairment (Ticinesi et al., 2019) and is also highly associated with obesity (Sze and Schloss, 2016). *Bacteroides*, one of the *Bacteroidetes*, can also be used as a biomarker for the recanalization of ischemic stroke (Chou et al., 2023) and the worsening of multiple sclerosis (Devolder et al., 2023), possibly because of the pathogenicity and pro-inflammatory neurotoxins it produces (Sears, 2009). *Enterobacteriaceae*, mostly considered as pro-inflammatory bacteria, have been shown to serve as noninvasive diagnostic biomarkers for Alzheimer’s disease (Chen et al., 2023) and epilepsy (Cui et al., 2021).

The transcriptomics of PSCI has also highlighted the effectiveness of certain miRNAs in terms of diagnosis, including miR-21, miR-132, miR-195, miR-200b, miR-497, and miR-let-7i. Thus far, research has validated the molecular diagnostic capability of miR-21 in several diseases such as acute cerebral infarction (Mohammed et al., 2022), atherosclerosis (Fontanella et al., 2021), and glioma (Zhou et al., 2018). Other studies have identified the diagnostic properties of miR-132 in mild cognitive impairment (Sheinerman et al., 2012; Xie et al., 2015) due to its modulation of glutamate receptor levels. Not only that, miR-132 also plays the role of a biomarker in the diagnosis of major depression and a variety of central nervous system disorders (van den Berg et al., 2020). The researchers also found miR-195 and miR-497 in acute stroke (Zhai et al., 2020) and cognitive impairment in schizophrenia (Huang et al., 2020). For miR-200b, current research has only identified its diagnostic possibilities in Alzheimer’s disease or mild cognitive impairment (Liu et al., 2014). And more study is still needed to fully understand the precise diagnostic performance of miR-let-7i.

Only neuroglobin, which has also been utilized in the diagnosis of delayed cerebral ischemia after craniocerebral injury (Chen et al., 2015) and subarachnoid hemorrhage (Ding et al., 2020), has been asserted as a diagnostic biomarker by the proteomics of PSCI. In contrast, the genomics of PSCI is limited to the study of candidate genes without biomarker discovery.

It is worth noting that only a small number of studies have demonstrated the diagnostic performance of these markers and their causal relationship with PSCI, such as neuroglobin (Gao et al., 2022), serum amyloid A (Zhang Y. et al., 2021), L-carnitine (Che et al., 2022) and TMAO (Zhu et al., 2020). The rest of the biomarkers have only shown associations with PSCI at a statistical level, and these changes may be related to multiple pathways including inflammation, so subsequent studies need to be approached with caution. But there is not enough evidence to show how these pathways interact with each other. Also, the studies that are already out there are still in the experimental stage and absence of any multicenter or large-sample cohort studies. This needs to be fixed in the future, which means that researchers will have to deal with results that aren’t identical because of different technologies, groups, or causes. Still, we prefer more study to be done on the omics of PSCI and how the results can be used in the clinic. More importantly, we need to come up with consistent diagnostic standards for these possible biomarkers so that we can quickly find the best ways to treat PSCI.

### 4.2 Possible mechanism

**Figure 3:**
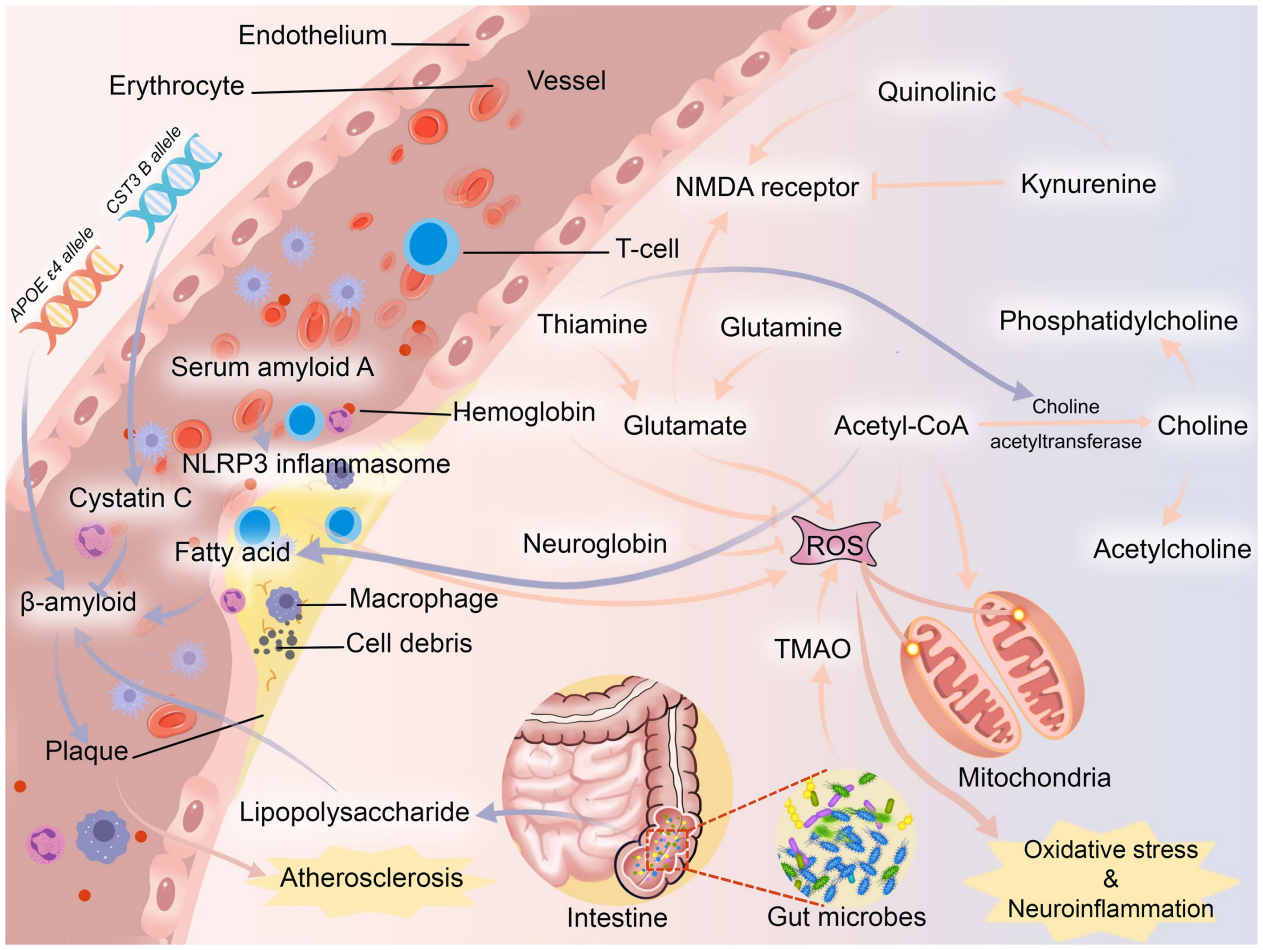
Possible pathogenesis of PSCI. NMDA receptor, N-methyl-D-aspartic acid receptor; NLRP3 inflammasome, NOD-like receptor thermal protein domain associated protein 3 inflammasome; TMAO, trimethylamine-n-oxide.

Inflammation and oxidative stress appear to play important roles in PSCI (Figure 3). It is well known that cognitive function is based on synaptic plasticity (Sloley et al., 2021). Thiamine, an antioxidant, binds to several mitochondrial enzymes and alters mitochondrial interactions (Marcé-Grau et al., 2019), and its deficiency reduces the level of the neurotransmitter acetylcholine by decreasing the activity of choline acetyltransferase (Jankowska-Kulawy et al., 2010) and induces excessive glutamate release (Mkrtchyan et al., 2016). Glutamine is a precursor of excitatory neurotransmitter glutamate, which plays an important role in synaptic plasticity (Reiner and Levitz, 2018). Overconsumption of glutamine can lead to mitochondrial damage and produce a large number of reactive oxygen species (ROS) (Verma et al., 2022), which induces neurotoxicity. Choline, a precursor of acetylcholine, is implicated in cholinergic transmission and signaling, whereas reduced or absent levels of choline in the brain lead to impaired cognitive function (de Medeiros et al., 2019). It is worth noting that choline is likewise recognized as a precursor to phosphatidylcholine, which slows cognitive decline (Blusztajn et al., 2017). An imbalance in the ratio of quinolinic acid, an NMDA (N-methyl-D-aspartic acid) receptor agonist, to kynurenine, an NMDA receptor antagonist, which is a kynurenine pathway metabolite of tryptophan and induces synaptic plasticity (Hestad et al., 2022), also contributes to neuroinflammation (Forrest et al., 2015). Also, hemoglobin and neuroglobin are neuroprotective, scavenging and detoxifying reactive oxygen species (Agyemang et al., 2021; Gorabi et al., 2021), so deficiencies in hemoglobin and neuroglobin predispose the body to cognitive dysfunction. In addition to these two proteins, serum amyloid A is involved in the chemotactic recruitment of inflammatory cells (Lee et al., 2020), and after stroke activates NLRP3 (NOD-like receptor thermal protein domain associated protein 3) inflammasome through oxidative stress to impair neuronal cells and cognitive function (Shridas and Tannock, 2019). The gut microbiota plays an important role in regulating the body’s metabolism, so ecological imbalances after stroke often cause activation of pro-inflammatory microglia leading to cognition-related neuroinflammation (Liu et al., 2022). Trimethylamine-n-oxide (TMAO) produced through gut microbial metabolism can lead to neuronal cell senescence by affecting mitochondrial energy metabolism, further exacerbating neuroinflammation and oxidative stress, ultimately leading to degeneration of brain function and cognitive impairment (Brunt et al., 2021).

Another important mechanism of PSCI may be atherosclerosis (Figure 3). After stroke, changes in the body’s fatty acid levels may lead to plaque formation by inducing the production of β-amyloid (Díaz et al., 2022). In turn, β-amyloid has neurotoxic effects on neurons (Fukuchi et al., 1993) and can be deposited in blood vessel walls leading to atherosclerosis (Stakos et al., 2020). The APOE ε4 allele was found to be a biologically active factor in β-amyloid deposition, (Rasmussen, 2016) and it is more likely to be found in post-stroke patients (Abboud et al., 2008). Even though β-amyloid may be initially eliminated by cystatin C, the neuroprotective effect of cystatin C decreases rapidly once the demand increases (Sheikh et al., 2021). In contrast, CST3 B, an allele of cystatin C, often leads to a decrease in cystatin C secretion, which ultimately leads to the development of atherosclerosis (Zeng et al., 2019). In addition, *Enterobacteriaceae*, one of the microorganisms, metabolizes lipopolysaccharide, which not only leads to the release of pro-inflammatory factors, but also leads to the deposition of β-amyloid (Płóciennikowska et al., 2015). Most animal studies have shown that β-amyloid is often deposited in the thalamus after stroke (van Groen et al., 2005; Mäkinen et al., 2008; Zhang J. et al., 2012), while other studies have found that β-amyloid is deposited in the hippocampus (Dietrich et al., 1998; Basak et al., 2023). It is well known that the thalamic nuclei play an important role in cognitive function (Dalrymple-Alford et al., 2015), and the hippocampus is closely related to the thalamic nuclei (Braak et al., 1996).

The main causes of PSCI tend to be neuroinflammation, oxidative stress, and atherosclerosis. Similar to Alzheimer’s disease (Acharya et al., 2024) and post-traumatic encephalopathy dementia (Kornblith et al., 2022), the pathophysiology of this disease remains complex and poorly understood. Existing research have primarily demonstrated a statistical link rather than a definitive causal relationship between specific mechanisms and the disease. Studies discovered that the accumulation of amyloid β might trigger an inflammatory reaction, and subsequent processes resulting from these inflammatory changes may be the underlying cause of the disease in PSCI (Lalancette-Hébert et al., 2012; Ott et al., 2018; Izzy et al., 2021). Likewise, oxidative stress can impact the integrity of neurons and the expression of genes, which in turn affects cognitive performance by causing changes in the structure of hippocampal dendrites (Chi et al., 2023). There is also data suggesting that atherosclerosis may be a separate yet collaborative disease phase of PSCI (Yang Z. et al., 2020). Thus far, ongoing research has not been able to conclusively establish the individual contribution of a specific mechanism in PSCI. Neuroinflammation, oxidative stress, and atherosclerosis are potential factors that could potentially contribute to the disease and/or its progression. However, the specific impact of these factors is still unknown. To fully comprehend the precise mechanisms and causal pathways underlying PSCI, more study is undoubtedly required in the future.

In addition, this paper focuses solely on the potential involvement of biomarkers in the pathogenesis within the omics context, without conducting a thorough assessment of current research findings. This limitation significantly hinders our understanding of the mechanism, which is a key drawback of the paper. Apart from these two possible mechanisms, cerebral small blood vessel disease (Teng et al., 2017), neuroanatomical lesions (Sun et al., 2014), and lymphatic pathway damage (Back et al., 2017) are also on the radar, but most of them are secondary to various levels of brain damage and lack a specific association with cognitive function. Based on this, we encourage scholars to comprehensively and further explore the pathogenesis of PSCI in order to solve the current clinical difficulties.

### 4.3 Feasible treatment and intervention

On the basis of existing omics research, we suggest treating PSCI with a combination of dietary intervention and drug-targeted therapy.

The main intervention for PSCI should be dietary changes since they are more widely accessible. ALA, EPA, and DHA are examples of *n*-3 unsaturated fatty acids, which are necessary fats that are exclusively found in food. *n*-3 unsaturated fatty acids (including ALA, EPA, and DHA) are essential fatty acids that can only be obtained through food. It has been found that *n*-3 fatty acids can produce specialized pro-resolving mediators through the cyclooxygenase and lipoxygenase pathways (Artiach et al., 2020; Christie and Harwood, 2020), while ALA and EPA have been shown to reduce the risk of neurological disorders such as stroke and mild cognitive impairment (Abdelhamid et al., 2020), and supplementation with ALA protects hippocampal neurons after a stroke and can also improve memory and spatial learning ability (Crupi et al., 2013). More than that, DHA has been found to reduce β-amyloid deposition and oxidative stress after stroke (Cardoso et al., 2016). Choline and its derivative betaine are involved in the irreversible cycling of methionine and homocysteine (Veskovic et al., 2019), and their addition to the diet may improve hyperhomocysteinemia to a certain extent (Rosas-Rodríguez and Valenzuela-Soto, 2021), thereby preventing stroke and cognitive impairment. L-carnitine supplementation has been found to not only improve cerebral blood flow supply in stroke patients (Endo et al., 2018) but also regulate mitochondrial energy metabolism and promote neurotransmitter release (Ferreira and McKenna, 2017), and about 3/4 of the body’s L-carnitine is obtained from the diet. Gut microbes can break down dietary fiber into a variety of short-chain fatty acids (SCFAs), which not only promote cognitive function but also provide synaptic plasticity and play an important role in the gut-brain axis (Dalile et al., 2019). Some studies have found that administration of SCFAs improved cognitive function and inhibited β-amyloid aggregation (Ho et al., 2018).

Pharmacological treatment is also required for PSCI. miRNAs exist in a stable form in human plasma and are not affected by endogenous RNase activity (Eisenberg et al., 2015). miRNAs in the blood of PSCI patients suffer from an imbalance in the miRNA cycle due to tissue damage (Pascual et al., 2021). Several studies have shown that miR-21 is not only an anti-apoptotic factor (Seike et al., 2009), but also has neuroprotective effects against cerebral ischemia/reperfusion injury, and moreover alleviates neuroinflammation (Yan et al., 2021). Whereas miR-132 has been found to be a key regulator of cognition (Walgrave et al., 2021), and overexpression inhibits learning ability (Cong et al., 2021). Drug therapy for PSCI can be targeted to regulate miRNA balance in the body, thereby regulating neuronal activity and maintaining synaptic plasticity. BDNF is also believed to regulate synaptic plasticity and enhance learning ability (Lu et al., 2014), and studies have shown that BDNF can promote the recovery of movement and sensation in stroke patients (Schäbitz et al., 2007). The treatment of BDNF can also be used as a new therapeutic target to improve PSCI in the future.

In order to implement targeted interventions at various stages of the disease’s incidence, we advise researchers to keep investigating the use of these biomarkers in PSCI intervention and therapy in the future and to provide constructive recommendations. However, looking at the future of PSCI intervention and treatment only from the perspective of omics is one of the limitations of this paper, because the current intervention and treatment initiatives for PSCI are numerous. The underlying method of ‘hierarchical prevention’ is more appropriate when discussing PSCI treatment and prevention from a non-omics standpoint. According to research, most dementia cases and strokes can be avoided (Casolla et al., 2019). We propose that primary preventive methods should take into account both stroke and cognitive impairment, since the pathophysiology of PSCI remains uncertain. Given the current body of data, early multi-target intervention based on lifestyle or vascular problems is imperative to reduce the occurrence of PSCI. High-risk factors for PSCI include genetic predisposition, vascular risk factors, and population variables (Huang et al., 2022), so treatment in the acute stage following a cerebrovascular injury is crucial. During secondary prevention phase, it is frequently essential to actively investigate the underlying cause and administer focused care, such as thrombus removal, hyperlipidemia control, education enhancement, and improved motor function training, to prevent stroke and early cognitive damage. Postponing the additional deterioration of cognitive function and enhancing daily living skills are the primary goals of tertiary prevention of PSCI. However, PSCI is now treated mostly with other cognitive dysfunction disorders, including drug therapy and non-drug therapy, as there are no large-scale, randomized, double-blind, controlled clinical trials available. Though further clinical research is required to validate their effectiveness in PSCI, recent studies have suggested that medications such as Actovegin (Guekht et al., 2017), *Ginkgo biloba* extract (Berthier et al., 2009), and Acetylcholinesterase inhibitors (Donepezil) (Kim et al., 2020) may be able to improve cognitive performance. Acupuncture (Wang et al., 2016), transcranial magnetic stimulation, transcranial direct current stimulation (Begemann et al., 2020), adaptive cognitive training (Tang et al., 2019), and other sophisticated non-pharmacological treatments for post-stroke cognitive impairment have all demonstrated some promise in enhancing cognitive function. Therefore, we are still looking forward to advances in the treatment of PSCI, whether based on omics or traditional hierarchical prevention, which may greatly improve patient outcomes.

## 5 Conclusion

All things considered, the use of biomarkers in mechanism mining, early diagnostic support, illness progression tracking, and therapeutic target investigations has been eye-opening. In the context of omics, uncovering potential biomarkers of disease, exploring molecular pathways of disease, and testing drug efficacy are no longer limited to the single analyses of the past, and advances in high-throughput technologies have made PSCI research less slow. It is also the demand for disease research that in return promotes the development of omics technology, and new technologies and new platforms continue to help PSCI research. However, omics studies of PSCI are in their infancy, and there are currently no approved biomarkers for the diagnosis and prediction of PSCI. Translating initial research into clinical applications requires a more rigorous validation process involving the use of expertise, the development of predictive models, and ethical and market regulation.

We believe that future omics-based studies will help to understand the specific interactions of these biomarkers, help to determine the meaning of changes at each time node and advance the study of PSCI-specific drugs. Omics will surely show a greater light in the future market, and its other potential applications are yet to be explored.

## Author contributions

QL: Writing – original draft, Investigation. AY: Writing – original draft. JP: Writing – review & editing, Data curation. DC: Writing – review & editing, Validation, Supervision. YZ: Writing – review & editing, Validation, Supervision. DB: Writing – review & editing, Supervision, Project administration, Funding acquisition. LY: Writing – review & editing, Conceptualization.

## References

[ref1] Aam S. Einstad M. S. Munthe-Kaas R. Lydersen S. Ihle-Hansen H. Knapskog A. B.. (2020). Post-stroke cognitive impairment-impact of follow-up time and stroke subtype on severity and cognitive profile: the nor-COAST study. Front. Neurol. 11:699. doi: 10.3389/fneur.2020.00699, PMID: 32765406 PMC7379332

[ref2] Abboud S. Viiri L. E. Lütjohann D. Goebeler S. Luoto T. Friedrichs S.. (2008). Associations of apolipoprotein E gene with ischemic stroke and intracranial atherosclerosis. Eur. J. Hum. Genet. 16, 955–960. doi: 10.1038/ejhg.2008.27, PMID: 18301447

[ref3] Abdelhamid A. S. Brown T. J. Brainard J. S. Biswas P. Thorpe G. C. Moore H. J.. (2020). Omega-3 fatty acids for the primary and secondary prevention of cardiovascular disease. Cochrane Database Syst. Rev. 3:Cd003177. doi: 10.1002/14651858.CD003177.pub5 32114706 PMC7049091

[ref4] Acharya N. K. Grossman H. C. Clifford P. M. Levin E. C. Light K. R. Choi H.. (2024). A chronic increase in blood-brain barrier permeability facilitates intraneuronal deposition of exogenous bloodborne amyloid-beta1-42 peptide in the brain and leads to Alzheimer’s disease-relevant cognitive changes in a mouse model. J. Alzheimers Dis. 98, 163–186. doi: 10.3233/JAD-231028, PMID: 38393907 PMC10977376

[ref5] Agyemang A. A. Kvist S. V. Brinkman N. Gentinetta T. Illa M. Ortenlöf N.. (2021). Cell-free oxidized hemoglobin drives reactive oxygen species production and pro-inflammation in an immature primary rat mixed glial cell culture. J. Neuroinflammation 18:42. doi: 10.1186/s12974-020-02052-4 33573677 PMC7879625

[ref6] Artiach G. Carracedo M. Plunde O. Wheelock C. E. Thul S. Sjövall P.. (2020). Omega-3 polyunsaturated fatty acids decrease aortic valve disease through the Resolvin E1 and ChemR23 axis. Circulation 142, 776–789. doi: 10.1161/CIRCULATIONAHA.119.041868, PMID: 32506925 PMC7439935

[ref7] Back D. B. Kwon K. J. Choi D. H. Shin C. Y. Lee J. Han S. H.. (2017). Chronic cerebral hypoperfusion induces post-stroke dementia following acute ischemic stroke in rats. J. Neuroinflammation 14:216. doi: 10.1186/s12974-017-0992-5 29121965 PMC5679180

[ref8] Baez E. Echeverria V. Cabezas R. Ávila-Rodriguez M. Garcia-Segura L. M. Barreto G. E. (2016). Protection by neuroglobin expression in brain pathologies. Front. Neurol. 7:146. doi: 10.3389/fneur.2016.00146 27672379 PMC5018480

[ref9] Bagyinszky E. Giau V. V. An S. A. (2020). Transcriptomics in Alzheimer’s disease: aspects and challenges. Int. J. Mol. Sci. 21:3517. doi: 10.3390/ijms21103517 32429229 PMC7278930

[ref10] Baierle M. Vencato P. H. Oldenburg L. Bordignon S. Zibetti M. Trentini C. M.. (2014). Fatty acid status and its relationship to cognitive decline and homocysteine levels in the elderly. Nutrients 6, 3624–3640. doi: 10.3390/nu6093624, PMID: 25221976 PMC4179179

[ref11] Balconi M. (2013). Dorsolateral prefrontal cortex, working memory and episodic memory processes: insight through transcranial magnetic stimulation techniques. Neurosci. Bull. 29, 381–389. doi: 10.1007/s12264-013-1309-z, PMID: 23385388 PMC5561838

[ref12] Barko P. C. McMichael M. A. Swanson K. S. Williams D. A. (2018). The gastrointestinal microbiome: a review. J. Vet. Intern. Med. 32, 9–25. doi: 10.1111/jvim.14875, PMID: 29171095 PMC5787212

[ref13] Basak J. M. Falk M. Mitchell D. N. Coakley K. A. Quillinan N. Orfila J. E.. (2023). Targeting BACE1-mediated production of amyloid beta improves hippocampal synaptic function in an experimental model of ischemic stroke. J. Cereb. Blood Flow Metab.:66:77. doi: 10.1177/0271678X231159597 PMC10638992 37150606

[ref14] Begemann M. J. Brand B. A. Ćurčić-Blake B. Aleman A. Sommer I. E. (2020). Efficacy of non-invasive brain stimulation on cognitive functioning in brain disorders: a meta-analysis. Psychol. Med. 50, 2465–2486. doi: 10.1017/S0033291720003670, PMID: 33070785 PMC7737055

[ref15] Berthier M. L. Green C. Lara J. P. Higueras C. Barbancho M. A. Dávila G.. (2009). Memantine and constraint-induced aphasia therapy in chronic poststroke aphasia. Ann. Neurol. 65, 577–585. doi: 10.1002/ana.21597, PMID: 19475666

[ref16] Bjerrum J. T. Nielsen O. H. Wang Y. L. Olsen J. (2008). Technology insight: metabonomics in gastroenterology-basic principles and potential clinical applications. Nat. Clin. Pract. Gastroenterol. Hepatol. 5, 332–343. doi: 10.1038/ncpgasthep1125, PMID: 18431374

[ref17] Blusztajn J. K. Slack B. E. Mellott T. J. (2017). Neuroprotective actions of dietary choline. Nutrients 9:8. doi: 10.3390/nu9080815 PMC5579609 28788094

[ref18] Braak H. Braak E. Yilmazer D. Bohl J. (1996). Functional anatomy of human hippocampal formation and related structures. J. Child Neurol. 11, 265–275. doi: 10.1177/088307389601100402, PMID: 8807415

[ref19] Brunt V. E. LaRocca T. J. Bazzoni A. E. Sapinsley Z. J. Miyamoto-Ditmon J. Gioscia-Ryan R. A.. (2021). The gut microbiome-derived metabolite trimethylamine N-oxide modulates neuroinflammation and cognitive function with aging. Geroscience 43, 377–394. doi: 10.1007/s11357-020-00257-2, PMID: 32862276 PMC8050157

[ref20] Cardoso C. Afonso C. Bandarra N. M. (2016). Dietary DHA and health: cognitive function ageing. Nutr. Res. Rev. 29, 281–294. doi: 10.1017/S0954422416000184 27866493

[ref21] Casolla B. Caparros F. Cordonnier C. Bombois S. Hénon H. Bordet R.. (2019). Biological and imaging predictors of cognitive impairment after stroke: a systematic review. J. Neurol. 266, 2593–2604. doi: 10.1007/s00415-018-9089-z, PMID: 30350168

[ref22] Castellanos M. Sobrino T. Pedraza S. Moldes O. Pumar J. M. Silva Y.. (2008). High plasma glutamate concentrations are associated with infarct growth in acute ischemic stroke. Neurology 71, 1862–1868. doi: 10.1212/01.wnl.0000326064.42186.7e 18971451

[ref23] Che B. Chen H. Wang A. Peng H. Bu X. Zhang J.. (2022). Association between plasma L-carnitine and cognitive impairment in patients with acute ischemic stroke. J. Alzheimers Dis. 86, 259–270. doi: 10.3233/JAD-215376, PMID: 35068454

[ref24] Chen H. Cao H. L. Chen S. W. Guo Y. Gao W. W. Tian H. L.. (2015). Neuroglobin and Nogo-a as biomarkers for the severity and prognosis of traumatic brain injury. Biomarkers 20, 495–501. doi: 10.3109/1354750X.2015.1094138 26472601

[ref25] Chen Z. Y. Patel P. D. Sant G. Meng C. X. Teng K. K. Hempstead B. L.. (2004). Variant brain-derived neurotrophic factor (BDNF) (Met66) alters the intracellular trafficking and activity-dependent secretion of wild-type BDNF in neurosecretory cells and cortical neurons. J. Neurosci. 24, 4401–4411. doi: 10.1523/JNEUROSCI.0348-04.2004, PMID: 15128854 PMC6729450

[ref26] Chen L. Wu J. (2012). Systems biology for complex diseases. J. Mol. Cell Biol. 4, 125–126. doi: 10.1093/jmcb/mjs022 22652391

[ref27] Chen G. Zhou X. Zhu Y. Shi W. Kong L. (2023). Gut microbiome characteristics in subjective cognitive decline, mild cognitive impairment and Alzheimer’s disease: a systematic review and meta-analysis. Eur. J. Neurol. 30, 3568–3580. doi: 10.1111/ene.15961, PMID: 37399128

[ref28] Chi X. Wang L. Liu H. Zhang Y. Shen W. (2023). Post-stroke cognitive impairment and synaptic plasticity: a review about the mechanisms and Chinese herbal drugs strategies. Front. Neurosci. 17:1123817. doi: 10.3389/fnins.2023.1123817, PMID: 36937659 PMC10014821

[ref29] Chou P. S. Hung W. C. Yang I. H. Kuo C. M. Wu M. N. Lin T. C.. (2023). Predicting adverse recanalization therapy outcomes in acute ischemic stroke patients using characteristic gut microbiota. Microorganisms 11:2016. doi: 10.3390/microorganisms11082016 37630576 PMC10458507

[ref30] Christie W. W. Harwood J. L. (2020). Oxidation of polyunsaturated fatty acids to produce lipid mediators. Essays Biochem. 64, 401–421. doi: 10.1042/EBC20190082, PMID: 32618335 PMC7517362

[ref31] Cogo A. Mangin G. Maïer B. Callebert J. Mazighi M. Chabriat H.. (2021). Increased serum QUIN/KYNA is a reliable biomarker of post-stroke cognitive decline. Mol. Neurodegener. 16:7. doi: 10.1186/s13024-020-00421-4 33588894 PMC7885563

[ref32] Cong L. Cong Y. Feng N. Liang W. Wu Y. (2021). Up-regulated microRNA-132 reduces the cognition-damaging effect of sevoflurane on Alzheimer’s disease rats by inhibiting FOXA1. Genomics 113, 3644–3652. doi: 10.1016/j.ygeno.2021.08.011, PMID: 34400241

[ref33] Crupi R. Marino A. Cuzzocrea S. (2013). *n*-3 fatty acids: role in neurogenesis and neuroplasticity. Curr. Med. Chem. 20, 2953–2963. doi: 10.2174/09298673113209990140, PMID: 23746276

[ref34] Cui G. Liu S. Liu Z. Chen Y. Wu T. Lou J.. (2021). Gut microbiome distinguishes patients with epilepsy from healthy individuals. Front. Microbiol. 12:696632. doi: 10.3389/fmicb.2021.696632 35069460 PMC8777111

[ref35] Dai X. Shen L. (2022). Advances and trends in omics technology development. Front. Med. 9:911861. doi: 10.3389/fmed.2022.911861, PMID: 35860739 PMC9289742

[ref36] Dalile B. Van Oudenhove L. Vervliet B. Verbeke K. (2019). The role of short-chain fatty acids in microbiota-gut-brain communication. Nat. Rev. Gastroenterol. Hepatol. 16, 461–478. doi: 10.1038/s41575-019-0157-3 31123355

[ref37] Dalrymple-Alford J. C. Harland B. Loukavenko E. A. Perry B. Mercer S. Collings D. A.. (2015). Anterior thalamic nuclei lesions and recovery of function: relevance to cognitive thalamus. Neurosci. Biobehav. Rev. 54, 145–160. doi: 10.1016/j.neubiorev.2014.12.007, PMID: 25637779

[ref38] Datta A. Chen C. Gao Y. G. Sze S. K. (2022). Quantitative proteomics of medium-sized extracellular vesicle-enriched plasma of lacunar infarction for the discovery of prognostic biomarkers. Int. J. Mol. Sci. 23:11670. doi: 10.3390/ijms231911670 36232970 PMC9569577

[ref39] de Medeiros L. M. De Bastiani M. A. Rico E. P. Schonhofen P. Pfaffenseller B. Wollenhaupt-Aguiar B.. (2019). Cholinergic differentiation of human neuroblastoma SH-SY5Y cell line and its potential use as an in vitro model for Alzheimer’s disease studies. Mol. Neurobiol. 56, 7355–7367. doi: 10.1007/s12035-019-1605-3, PMID: 31037648

[ref40] Devolder L. Pauwels A. Van Remoortel A. Falony G. Vieira-Silva S. Nagels G.. (2023). Gut microbiome composition is associated with long-term disability worsening in multiple sclerosis. Gut Microbes 15:2180316. doi: 10.1080/19490976.2023.2180316 36803643 PMC9980703

[ref41] Díaz G. Lengele L. Sourdet S. Soriano G. de Souto Barreto P. (2022). Nutrients and amyloid β status in the brain: a narrative review. Ageing Res. Rev. 81:101728. doi: 10.1016/j.arr.2022.101728, PMID: 36049590

[ref42] Dietrich W. D. Kraydieh S. Prado R. Stagliano N. E. (1998). White matter alterations following thromboembolic stroke: a beta-amyloid precursor protein immunocytochemical study in rats. Acta Neuropathol. 95, 524–531. doi: 10.1007/s004010050833, PMID: 9600599

[ref43] Ding C. Kang D. Chen P. Wang Z. Lin Y. Wang D.. (2020). Early stage neuroglobin level as a predictor of delayed cerebral ischemia in patients with aneurysmal subarachnoid hemorrhage. Brain Behav. 10:e01547. doi: 10.1002/brb3.1547 32026621 PMC7066341

[ref44] Dirnagl U. Iadecola C. Moskowitz M. A. (1999). Pathobiology of ischaemic stroke: an integrated view. Trends Neurosci. 22, 391–397. doi: 10.1016/S0166-2236(99)01401-0, PMID: 10441299

[ref45] Domon B. Aebersold R. (2006). Mass spectrometry and protein analysis. Science 312, 212–217. doi: 10.1126/science.1124619 16614208

[ref46] Douiri A. Rudd A. G. Wolfe C. D. (2013). Prevalence of poststroke cognitive impairment: South London stroke register 1995–2010. Stroke 44, 138–145. doi: 10.1161/STROKEAHA.112.670844 23150656

[ref47] Durga J. van Boxtel M. P. Schouten E. G. Kok F. J. Jolles J. Katan M. B.. (2007). Effect of 3-year folic acid supplementation on cognitive function in older adults in the FACIT trial: a randomised, double blind, controlled trial. Lancet 369, 208–216. doi: 10.1016/S0140-6736(07)60109-3 17240287

[ref48] Eisenberg I. Kotaja N. Goldman-Wohl D. Imbar T. (2015). microRNA in human reproduction. Adv. Exp. Med. Biol. 888, 353–387. doi: 10.1007/978-3-319-22671-2_18 26663192

[ref49] Endo S. Takahashi T. Sato M. Noya Y. Obana M. (2018). Effects of l-carnitine supplementation, botulinum neurotoxin injection, and rehabilitation for a chronic stroke patient. J. Stroke Cerebrovasc. Dis. 27, 3342–3344. doi: 10.1016/j.jstrokecerebrovasdis.2018.07.033, PMID: 30181037

[ref50] Estrada Sánchez A. M. Mejía-Toiber J. Massieu L. (2008). Excitotoxic neuronal death and the pathogenesis of Huntington’s disease. Arch. Med. Res. 39, 265–276. doi: 10.1016/j.arcmed.2007.11.011 18279698

[ref51] Feng L. He W. Huang G. Lin S. Yuan C. Cheng H.. (2020). Reduced thiamine is a predictor for cognitive impairment of cerebral infarction. Brain Behav. 10:e01709. doi: 10.1002/brb3.1709 32755028 PMC7507112

[ref52] Ferreira G. C. McKenna M. C. (2017). L-carnitine and acetyl-L-carnitine roles and neuroprotection in developing brain. Neurochem. Res. 42, 1661–1675. doi: 10.1007/s11064-017-2288-7, PMID: 28508995 PMC5621476

[ref53] Fiehn O. (2002). Metabolomics—the link between genotypes and phenotypes. Plant Mol. Biol. 48, 155–171. doi: 10.1023/A:1013713905833 11860207

[ref54] Fontanella R. A. Scisciola L. Rizzo M. R. Surina S. Sardu C. Marfella R.. (2021). Adiponectin related vascular and cardiac benefits in obesity: is there a role for an epigenetically regulated mechanism? Front. Cardiovasc. Med. 8:768026. doi: 10.3389/fcvm.2021.768026, PMID: 34869683 PMC8639875

[ref55] Forrest C. M. McNair K. Pisar M. Khalil O. S. Darlington L. G. Stone T. W. (2015). Altered hippocampal plasticity by prenatal kynurenine administration, kynurenine-3-monoxygenase (KMO) deletion or galantamine. Neuroscience 310, 91–105. doi: 10.1016/j.neuroscience.2015.09.022, PMID: 26365611 PMC4642643

[ref56] Fu W. J. Stromberg A. J. Viele K. Carroll R. J. Wu G. (2010). Statistics and bioinformatics in nutritional sciences: analysis of complex data in the era of systems biology. J. Nutr. Biochem. 21, 561–572. doi: 10.1016/j.jnutbio.2009.11.007 20233650 PMC2885517

[ref57] Fukuchi K. Sopher B. Martin G. M. (1993). Neurotoxicity of beta-amyloid. Nature 361, 122–123. doi: 10.1038/361122a0 8421518

[ref58] Gallo S. Sala V. Gatti S. Crepaldi T. (2015). Cellular and molecular mechanisms of HGF/met in the cardiovascular system. Clin. Sci. 129, 1173–1193. doi: 10.1042/CS20150502 26561593

[ref59] Gao Y. Wang B. Miao Y. Han Y. (2022). Serum Neuroglobin as a potential prognostic biomarker for cognitive impairment after intracerebral hemorrhage. Front. Neurol. 13:885323. doi: 10.3389/fneur.2022.885323, PMID: 35463129 PMC9021832

[ref60] Gaynor E. Rohde D. Large M. Mellon L. Hall P. Brewer L.. (2018). Cognitive impairment, vulnerability, and mortality post ischemic stroke: a five-year follow-up of the action on secondary prevention interventions and rehabilitation in stroke (ASPIRE-S) cohort. J. Stroke Cerebrovasc. Dis. 27, 2466–2473. doi: 10.1016/j.jstrokecerebrovasdis.2018.05.002, PMID: 29803601

[ref61] Giri M. Zhang M. Lü Y. (2016). Genes associated with Alzheimer’s disease: an overview and current status. Clin. Interv. Aging 11, 665–681. doi: 10.2147/CIA.S105769, PMID: 27274215 PMC4876682

[ref62] Godin O. Tzourio C. Maillard P. Alpérovitch A. Mazoyer B. Dufouil C. (2009). Apolipoprotein E genotype is related to progression of white matter lesion load. Stroke 40, 3186–3190. doi: 10.1161/STROKEAHA.109.555839, PMID: 19644067

[ref63] Gold A. B. Herrmann N. Swardfager W. Black S. E. Aviv R. I. Tennen G.. (2011). The relationship between indoleamine 2,3-dioxygenase activity and post-stroke cognitive impairment. J. Neuroinflammation 8:17. doi: 10.1186/1742-2094-8-17, PMID: 21324164 PMC3055827

[ref64] Gong L. Wang H. Zhu X. Dong Q. Yu Q. Mao B.. (2021). Nomogram to predict cognitive dysfunction after a minor ischemic stroke in hospitalized-population. Front. Aging Neurosci. 13:637363. doi: 10.3389/fnagi.2021.637363, PMID: 33967738 PMC8098660

[ref65] Gorabi A. M. Aslani S. Barreto G. E. Báez-Jurado E. Kiaie N. Jamialahmadi T.. (2021). The potential of mitochondrial modulation by neuroglobin in treatment of neurological disorders. Free Radic. Biol. Med. 162, 471–477. doi: 10.1016/j.freeradbiomed.2020.11.002, PMID: 33166649

[ref66] Guekht A. Skoog I. Edmundson S. Zakharov V. Korczyn A. D. (2017). ARTEMIDA trial (a randomized trial of efficacy, 12 months international double-blind actovegin): a randomized controlled trial to assess the efficacy of actovegin in poststroke cognitive impairment. Stroke 48, 1262–1270. doi: 10.1161/STROKEAHA.116.014321, PMID: 28432265 PMC5404405

[ref67] Guillemin G. J. (2012). Quinolinic acid, the inescapable neurotoxin. FEBS J. 279, 1356–1365. doi: 10.1111/j.1742-4658.2012.08485.x, PMID: 22248144

[ref68] Gurol M. E. Irizarry M. C. Smith E. E. Raju S. Diaz-Arrastia R. Bottiglieri T.. (2006). Plasma beta-amyloid and white matter lesions in AD, MCI, and cerebral amyloid angiopathy. Neurology 66, 23–29. doi: 10.1212/01.wnl.0000191403.95453.6a, PMID: 16401840

[ref69] Han Z. Qi L. Xu Q. Xu M. Cai L. Wong J.. (2020). BDNF met allele is associated with lower cognitive function in poststroke rehabilitation. Neurorehabil. Neural Repair 34, 247–259. doi: 10.1177/1545968320902127, PMID: 32009534

[ref70] Han Y. Zhou A. Li F. Wang Q. Xu L. Jia J. (2020). Apolipoprotein E epsilon4 allele is associated with vascular cognitive impairment no dementia in Chinese population. J. Neurol. Sci. 409:116606. doi: 10.1016/j.jns.2019.116606, PMID: 31865187

[ref71] Hansen K. F. Karelina K. Sakamoto K. Wayman G. A. Impey S. Obrietan K. (2013). miRNA-132: a dynamic regulator of cognitive capacity. Brain Struct. Funct. 218, 817–831. doi: 10.1007/s00429-012-0431-4, PMID: 22706759 PMC3508255

[ref72] Hazelwood H. S. Frank J. A. Maglinger B. McLouth C. J. Trout A. L. Turchan-Cholewo J.. (2022). Plasma protein alterations during human large vessel stroke: a controlled comparison study. Neurochem. Int. 160:105421. doi: 10.1016/j.neuint.2022.105421, PMID: 36179808

[ref73] Heisler J. M. O’Connor J. C. (2015). Indoleamine 2,3-dioxygenase-dependent neurotoxic kynurenine metabolism mediates inflammation-induced deficit in recognition memory. Brain Behav. Immun. 50, 115–124. doi: 10.1016/j.bbi.2015.06.022, PMID: 26130057 PMC4631688

[ref74] Hestad K. Alexander J. Rootwelt H. Aaseth J. O. (2022). The role of tryptophan dysmetabolism and quinolinic acid in depressive and neurodegenerative diseases. Biomolecules 12:998. doi: 10.3390/biom12070998 35883554 PMC9313172

[ref75] Ho L. Ono K. Tsuji M. Mazzola P. Singh R. Pasinetti G. M. (2018). Protective roles of intestinal microbiota derived short chain fatty acids in Alzheimer’s disease-type beta-amyloid neuropathological mechanisms. Expert. Rev. Neurother. 18, 83–90. doi: 10.1080/14737175.2018.1400909, PMID: 29095058 PMC5958896

[ref76] Huang X. Bao C. Lv Q. Zhao J. Wang Y. Lang X.. (2020). Sex difference in cognitive impairment in drug-free schizophrenia: association with miR-195 levels. Psychoneuroendocrinology 119:104748. doi: 10.1016/j.psyneuen.2020.104748, PMID: 32559610

[ref77] Huang Y. Y. Chen S. D. Leng X. Y. Kuo K. Wang Z. T. Cui M.. (2022). Post-stroke cognitive impairment: epidemiology, risk factors, and management. J. Alzheimers Dis. 86, 983–999. doi: 10.3233/JAD-215644, PMID: 35147548

[ref78] Huang Y. Shen Z. He W. (2021). Identification of gut microbiome signatures in patients with post-stroke cognitive impairment and affective disorder. Front. Aging Neurosci. 13:706765. doi: 10.3389/fnagi.2021.706765, PMID: 34489677 PMC8417941

[ref79] Huang S. Zhao J. Huang D. Zhuo L. Liao S. Jiang Z. (2016). Serum miR-132 is a risk marker of post-stroke cognitive impairment. Neurosci. Lett. 615, 102–106. doi: 10.1016/j.neulet.2016.01.028, PMID: 26806865

[ref80] Izzy S. Brown-Whalen A. Yahya T. Sarro-Schwartz A. Jin G. Chung J. Y.. (2021). Repetitive traumatic brain injury causes neuroinflammation before tau pathology in adolescent P301S mice. Int. J. Mol. Sci. 22:907. doi: 10.3390/ijms22020907 33477535 PMC7831108

[ref81] Jankowska-Kulawy A. Bielarczyk H. Pawełczyk T. Wróblewska M. Szutowicz A. (2010). Acetyl-CoA and acetylcholine metabolism in nerve terminal compartment of thiamine deficient rat brain. J. Neurochem. 115, 333–342. doi: 10.1111/j.1471-4159.2010.06919.x, PMID: 20649840

[ref82] Kaeser S. A. Herzig M. C. Coomaraswamy J. Kilger E. Selenica M. L. Winkler D. T.. (2007). Cystatin C modulates cerebral beta-amyloidosis. Nat. Genet. 39, 1437–1439. doi: 10.1038/ng.2007.23 18026102

[ref83] Kekuda R. Manoharan P. Baseler W. Sundaram U. (2013). Monocarboxylate 4 mediated butyrate transport in a rat intestinal epithelial cell line. Dig. Dis. Sci. 58, 660–667. doi: 10.1007/s10620-012-2407-x, PMID: 23344966

[ref84] Kim J. O. Lee S. J. Pyo J. S. (2020). Effect of acetylcholinesterase inhibitors on post-stroke cognitive impairment and vascular dementia: a meta-analysis. PLoS One 15:e0227820. doi: 10.1371/journal.pone.0227820 32032361 PMC7006920

[ref85] Kim K. Y. Shin K. Y. Chang K. A. (2022). Potential biomarkers for post-stroke cognitive impairment: a systematic review and meta-analysis. Int. J. Mol. Sci. 23:602. doi: 10.3390/ijms23020602 35054785 PMC8775398

[ref86] Kim J. M. Stewart R. Park M. S. Kang H. J. Kim S. W. Shin I. S.. (2012). Associations of BDNF genotype and promoter methylation with acute and long-term stroke outcomes in an east Asian cohort. PLoS One 7:e51280. doi: 10.1371/journal.pone.0051280 23240009 PMC3519835

[ref87] Kisilevsky R. Manley P. N. (2012). Acute-phase serum amyloid a: perspectives on its physiological and pathological roles. Amyloid 19, 5–14. doi: 10.3109/13506129.2011.654294, PMID: 22320226

[ref88] Kornblith E. Bahorik A. Li Y. Peltz C. B. Barnes D. E. Yaffe K. (2022). Traumatic brain injury, cardiovascular disease, and risk of dementia among older US veterans. Brain Inj. 36, 628–632. doi: 10.1080/02699052.2022.2033842, PMID: 35099335 PMC9187591

[ref89] Kotlęga D. Peda B. Drozd A. Zembroń-Łacny A. Stachowska E. Gramacki J.. (2021a). Prostaglandin E2, 9S-, 13S-HODE and resolvin D1 are strongly associated with the post-stroke cognitive impairment. Prostaglandins Other Lipid Mediat. 156:106576. doi: 10.1016/j.prostaglandins.2021.106576, PMID: 34119645

[ref90] Kotlęga D. Peda B. Palma J. Zembroń-Łacny A. Gołąb-Janowska M. Masztalewicz M.. (2021b). Free fatty acids are associated with the cognitive functions in stroke survivors. Int. J. Environ. Res. Public Health 18:12. doi: 10.3390/ijerph18126500 PMC8296333 34208689

[ref91] Kotlega D. Zembron-Lacny A. Morawin B. Golab-Janowska M. Nowacki P. Szczuko M. (2020). Free fatty acids and their inflammatory derivatives affect BDNF in stroke patients. Mediat. Inflamm. 2020, 1–12. doi: 10.1155/2020/6676247 PMC7728491 33343231

[ref92] Kroll M. H. Harris T. S. Moake J. L. Handin R. I. Schafer A. I. (1991). von Willebrand factor binding to platelet GpIb initiates signals for platelet activation. J. Clin. Invest. 88, 1568–1573. doi: 10.1172/JCI115468, PMID: 1939645 PMC295673

[ref93] Kurozumi K. Nakamura K. Tamiya T. Kawano Y. Kobune M. Hirai S.. (2004). BDNF gene-modified mesenchymal stem cells promote functional recovery and reduce infarct size in the rat middle cerebral artery occlusion model. Mol. Ther. 9, 189–197. doi: 10.1016/j.ymthe.2003.10.012, PMID: 14759803

[ref94] Lalancette-Hébert M. Swarup V. Beaulieu J. M. Bohacek I. Abdelhamid E. Weng Y. C.. (2012). Galectin-3 is required for resident microglia activation and proliferation in response to ischemic injury. J. Neurosci. 32, 10383–10395. doi: 10.1523/JNEUROSCI.1498-12.2012, PMID: 22836271 PMC6703730

[ref95] Layeghifard M. Hwang D. M. Guttman D. S. (2017). Disentangling interactions in the microbiome: a network perspective. Trends Microbiol. 25, 217–228. doi: 10.1016/j.tim.2016.11.008, PMID: 27916383 PMC7172547

[ref96] Lee J. Y. Hall J. A. Kroehling L. Wu L. Najar T. Nguyen H. H.. (2020). Serum amyloid a proteins induce pathogenic Th17 cells and promote inflammatory disease. Cell 180, 79–91.e16. doi: 10.1016/j.cell.2019.11.026, PMID: 31866067 PMC7039443

[ref97] Lee E. J. Kim D. J. Kang D. W. Yang W. Jeong H. Y. Kim J. M.. (2023). Targeted Metabolomic biomarkers for stroke subtyping. Transl. Stroke Res. 15, 422–432. doi: 10.1007/s12975-023-01137-5 36764997

[ref98] Lee S. C. Lee K. Y. Kim Y. J. Kim S. H. Koh S. H. Lee Y. J. (2010). Serum VEGF levels in acute ischaemic strokes are correlated with long-term prognosis. Eur. J. Neurol. 17, 45–51. doi: 10.1111/j.1468-1331.2009.02731.x, PMID: 19566899

[ref99] Levy E. Sastre M. Kumar A. Gallo G. Piccardo P. Ghetti B.. (2001). Codeposition of cystatin C with amyloid-beta protein in the brain of Alzheimer disease patients. J. Neuropathol. Exp. Neurol. 60, 94–104. doi: 10.1093/jnen/60.1.94, PMID: 11202179

[ref100] Li E. H. Huang Q. Z. Li G. C. Xiang Z. Y. Zhang X. (2017). Effects of miRNA-200b on the development of diabetic retinopathy by targeting *VEGFA* gene. Biosci. Rep. 37:BSR20160572. doi: 10.1042/BSR20160572 28122882 PMC5484021

[ref101] Li W. Shao C. Zhou H. Du H. Chen H. Wan H.. (2022). Multi-omics research strategies in ischemic stroke: a multidimensional perspective. Ageing Res. Rev. 81:101730. doi: 10.1016/j.arr.2022.101730, PMID: 36087702

[ref102] Li Z. Zhu M. Meng C. Lin H. Huang L. (2022). Predictive value of serum adiponectin and hemoglobin levels for vascular cognitive impairment in ischemic stroke patients. *Pak*. J. Med. Sci. 38, 705–710. doi: 10.12669/pjms.38.3.5204 PMC9002452 35480518

[ref103] Ling Y. Gong T. Zhang J. Gu Q. Gao X. Weng X.. (2020a). Gut microbiome signatures are biomarkers for cognitive impairment in patients with ischemic stroke. Front. Aging Neurosci. 12:511562. doi: 10.3389/fnagi.2020.511562, PMID: 33192448 PMC7645221

[ref104] Ling Y. Gu Q. Zhang J. Gong T. Weng X. Liu J.. (2020b). Structural change of gut microbiota in patients with post-stroke comorbid cognitive impairment and depression and its correlation with clinical features. J. Alzheimers Dis. 77, 1595–1608. doi: 10.3233/JAD-200315, PMID: 32925035

[ref105] Liu T. W. Chen C. M. Chang K. H. (2022). Biomarker of neuroinflammation in Parkinson’s disease. Int. J. Mol. Sci. 23:4148. doi: 10.3390/ijms23084148 35456966 PMC9028544

[ref106] Liu Y. Kong C. Gong L. Zhang X. Zhu Y. Wang H.. (2020a). The association of post-stroke cognitive impairment and gut microbiota and its corresponding metabolites. J. Alzheimers Dis. 73, 1455–1466. doi: 10.3233/JAD-191066, PMID: 31929168

[ref107] Liu Y. Wang S. Kan J. Zhang J. Zhou L. Huang Y.. (2020b). Chinese herbal medicine interventions in neurological disorder therapeutics by regulating glutamate signaling. Curr. Neuropharmacol. 18, 260–276. doi: 10.2174/1570159X17666191101125530, PMID: 31686629 PMC7327939

[ref108] Liu C. G. Wang J. L. Li L. Xue L. X. Zhang Y. Q. Wang P. C. (2014). MicroRNA-135a and -200b, potential biomarkers for Alzheimer’s disease, regulate β secretase and amyloid precursor protein. Brain Res. 1583, 55–64. doi: 10.1016/j.brainres.2014.04.026, PMID: 25152461

[ref109] Liu M. Zhou K. Li H. Dong X. Tan G. Chai Y.. (2015). Potential of serum metabolites for diagnosing post-stroke cognitive impairment. Mol. Biosyst. 11, 3287–3296. doi: 10.1039/C5MB00470E, PMID: 26490688

[ref110] Lo J. W. Crawford J. D. Desmond D. W. Godefroy O. Jokinen H. Mahinrad S.. (2019). Profile of and risk factors for poststroke cognitive impairment in diverse ethnoregional groups. Neurology 93, e2257–e2271. doi: 10.1212/WNL.0000000000008612, PMID: 31712368 PMC6937495

[ref111] Lockhart D. J. Winzeler E. A. (2000). Genomics, gene expression and DNA arrays. Nature 405, 827–836. doi: 10.1038/35015701, PMID: 10866209

[ref112] Lopez I. A. Acuna D. Shahram Y. Mowlds D. Ngan A. M. Rungvivatjarus T.. (2010). Neuroglobin expression in the cochlea of rat pups exposed to chronic very mild carbon monoxide (25 ppm) in air during and after the prenatal period. Brain Res. 1327, 56–68. doi: 10.1016/j.brainres.2010.02.078 20211612

[ref113] Lopez M. S. Dempsey R. J. Vemuganti R. (2017). The microRNA miR-21 conditions the brain to protect against ischemic and traumatic injuries. Cond. Med. 1, 35–46, PMID: 34268484 PMC8279043

[ref114] Lozupone C. A. Stombaugh J. I. Gordon J. I. Jansson J. K. Knight R. (2012). Diversity, stability and resilience of the human gut microbiota. Nature 489, 220–230. doi: 10.1038/nature11550, PMID: 22972295 PMC3577372

[ref115] Lu B. (2003). BDNF and activity-dependent synaptic modulation. Learn. Mem. 10, 86–98. doi: 10.1101/lm.54603 12663747 PMC5479144

[ref116] Lu B. Nagappan G. Lu Y. (2014). BDNF and synaptic plasticity, cognitive function, and dysfunction. Handb. Exp. Pharmacol. 220, 223–250. doi: 10.1007/978-3-642-45106-5_9 24668475

[ref117] Mäkinen S. van Groen T. Clarke J. Thornell A. Corbett D. Hiltunen M.. (2008). Coaccumulation of calcium and beta-amyloid in the thalamus after transient middle cerebral artery occlusion in rats. J. Cereb. Blood Flow Metab. 28, 263–268. doi: 10.1038/sj.jcbfm.9600529, PMID: 17653130

[ref118] Marcé-Grau A. Martí-Sánchez L. Baide-Mairena H. Ortigoza-Escobar J. D. Pérez-Dueñas B. (2019). Genetic defects of thiamine transport and metabolism: a review of clinical phenotypes, genetics, and functional studies. J. Inherit. Metab. Dis. 42, 581–597. doi: 10.1002/jimd.12125, PMID: 31095747

[ref119] Meng N. Shi S. Su Y. (2016). Proton magnetic resonance spectroscopy as a diagnostic biomarker in mild cognitive impairment following stroke in acute phase. Neuroreport 27, 559–563. doi: 10.1097/WNR.0000000000000555, PMID: 26981713

[ref120] Mijajlović M. D. Pavlović A. Brainin M. Heiss W. D. Quinn T. J. Ihle-Hansen H. B.. (2017). Post-stroke dementia—a comprehensive review. BMC Med. 15:11. doi: 10.1186/s12916-017-0779-7 28095900 PMC5241961

[ref121] Mkrtchyan G. Graf A. Bettendorff L. Bunik V. (2016). Cellular thiamine status is coupled to function of mitochondrial 2-oxoglutarate dehydrogenase. Neurochem. Int. 101, 66–75. doi: 10.1016/j.neuint.2016.10.009, PMID: 27773789

[ref122] Mohammed A. Shaker O. G. Khalil M. A. F. Gomaa M. Fathy S. A. Abu-El-Azayem A. K.. (2022). Long non-coding RNA NBAT1, TUG1, miRNA-335, and miRNA-21 as potential biomarkers for acute ischemic stroke and their possible correlation to thyroid hormones. Front. Mol. Biosci. 9:914506. doi: 10.3389/fmolb.2022.914506, PMID: 36250025 PMC9565477

[ref123] Nicholson J. K. Lindon J. C. Holmes E. (1999). ‘Metabonomics’: understanding the metabolic responses of living systems to pathophysiological stimuli via multivariate statistical analysis of biological NMR spectroscopic data. Xenobiotica 29, 1181–1189. doi: 10.1080/004982599238047, PMID: 10598751

[ref124] Niendam T. A. Laird A. R. Ray K. L. Dean Y. M. Glahn D. C. Carter C. S. (2012). Meta-analytic evidence for a superordinate cognitive control network subserving diverse executive functions. Cogn. Affect. Behav. Neurosci. 12, 241–268. doi: 10.3758/s13415-011-0083-5, PMID: 22282036 PMC3660731

[ref125] Ott B. R. Jones R. N. Daiello L. A. de la Monte S. M. Stopa E. G. Johanson C. E.. (2018). Blood-cerebrospinal fluid barrier gradients in mild cognitive impairment and Alzheimer’s disease: relationship to inflammatory cytokines and chemokines. Front. Aging Neurosci. 10:245. doi: 10.3389/fnagi.2018.00245, PMID: 30186149 PMC6110816

[ref126] Pascoe M. C. Linden T. (2016). Folate and MMA predict cognitive impairment in elderly stroke survivors: a cross sectional study. Psychiatry Res. 243, 49–52. doi: 10.1016/j.psychres.2016.06.008, PMID: 27367490

[ref127] Pascual M. Calvo-Rodriguez M. Núñez L. Villalobos C. Ureña J. Guerri C. (2021). Toll-like receptors in neuroinflammation, neurodegeneration, and alcohol-induced brain damage. IUBMB Life 73, 900–915. doi: 10.1002/iub.2510, PMID: 34033211

[ref128] Płóciennikowska A. Hromada-Judycka A. Borzęcka K. Kwiatkowska K. (2015). Co-operation of TLR4 and raft proteins in LPS-induced pro-inflammatory signaling. Cell. Mol. Life Sci. 72, 557–581. doi: 10.1007/s00018-014-1762-5, PMID: 25332099 PMC4293489

[ref129] Potter T. Lioutas V. A. Tano M. Pan A. Meeks J. Woo D.. (2021). Cognitive impairment after intracerebral hemorrhage: a systematic review of current evidence and knowledge gaps. Front. Neurol. 12:716632. doi: 10.3389/fneur.2021.716632, PMID: 34512528 PMC8429504

[ref130] Prodjohardjono A. Vidyanti A. N. Susianti N. A. Sudarmanta Sutarni S. Setyopranoto I. (2020). Higher level of acute serum VEGF and larger infarct volume are more frequently associated with post-stroke cognitive impairment. PLoS One 15:e0239370. doi: 10.1371/journal.pone.0239370 33017430 PMC7535035

[ref131] Qi B. Kong L. Lai X. Wang L. Liu F. Ji W.. (2023). Plasma exosome proteomics reveals the pathogenesis mechanism of post-stroke cognitive impairment. Aging 15, 4334–4362. doi: 10.18632/aging.204738, PMID: 37211381 PMC10258006

[ref132] Quince C. Walker A. W. Simpson J. T. Loman N. J. Segata N. (2017). Shotgun metagenomics, from sampling to analysis. Nat. Biotechnol. 35, 833–844. doi: 10.1038/nbt.3935 28898207

[ref133] Rasmussen K. L. (2016). Plasma levels of apolipoprotein E, APOE genotype and risk of dementia and ischemic heart disease: a review. Atherosclerosis 255, 145–155. doi: 10.1016/j.atherosclerosis.2016.10.037, PMID: 28340945

[ref134] Reiner A. Levitz J. (2018). Glutamatergic signaling in the central nervous system: ionotropic and metabotropic receptors in concert. Neuron 98, 1080–1098. doi: 10.1016/j.neuron.2018.05.018, PMID: 29953871 PMC6484838

[ref135] Rezaei S. Asgari Mobarake K. Saberi A. Keshavarz P. Leili E. K. (2016). Brain-derived neurotrophic factor (BDNF) Val66Met polymorphism and post-stroke dementia: a hospital-based study from northern Iran. Neurol. Sci. 37, 935–942. doi: 10.1007/s10072-016-2520-2, PMID: 27071687

[ref136] Ribas G. S. Vargas C. R. Wajner M. (2014). L-carnitine supplementation as a potential antioxidant therapy for inherited neurometabolic disorders. Gene 533, 469–476. doi: 10.1016/j.gene.2013.10.017, PMID: 24148561

[ref137] Rohlff C. (2001). Proteomics in neuropsychiatric disorders. Int. J. Neuropsychopharmacol. 4, 93–102. doi: 10.1017/S1461145701002267, PMID: 11343634

[ref138] Rosas-Rodríguez J. A. Valenzuela-Soto E. M. (2021). The glycine betaine role in neurodegenerative, cardiovascular, hepatic, and renal diseases: insights into disease and dysfunction networks. Life Sci. 285:119943. doi: 10.1016/j.lfs.2021.119943 34516992

[ref139] Sack G. H. Jr. (2018). Serum amyloid A—a review. Mol. Med. 24:46. doi: 10.1186/s10020-018-0047-0 30165816 PMC6117975

[ref140] Sancesario G. M. Bernardini S. (2018). Alzheimer’s disease in the omics era. Clin. Biochem. 59, 9–16. doi: 10.1016/j.clinbiochem.2018.06.011, PMID: 29920246

[ref141] Santamaría A. Galván-Arzate S. Lisý V. Ali S. F. Duhart H. M. Osorio-Rico L.. (2001). Quinolinic acid induces oxidative stress in rat brain synaptosomes. Neuroreport 12, 871–874. doi: 10.1097/00001756-200103260-00049, PMID: 11277599

[ref142] Sapko M. T. Guidetti P. Yu P. Tagle D. A. Pellicciari R. Schwarcz R. (2006). Endogenous kynurenate controls the vulnerability of striatal neurons to quinolinate: implications for Huntington’s disease. Exp. Neurol. 197, 31–40. doi: 10.1016/j.expneurol.2005.07.004, PMID: 16099455

[ref143] Sastre M. Calero M. Pawlik M. Mathews P. M. Kumar A. Danilov V.. (2004). Binding of cystatin C to Alzheimer’s amyloid beta inhibits in vitro amyloid fibril formation. Neurobiol. Aging 25, 1033–1043. doi: 10.1016/j.neurobiolaging.2003.11.006, PMID: 15212828

[ref144] Schäbitz W. R. Steigleder T. Cooper-Kuhn C. M. Schwab S. Sommer C. Schneider A.. (2007). Intravenous brain-derived neurotrophic factor enhances poststroke sensorimotor recovery and stimulates neurogenesis. Stroke 38, 2165–2172. doi: 10.1161/STROKEAHA.106.477331, PMID: 17510456

[ref145] Schweiger J. I. Bilek E. Schäfer A. Braun U. Moessnang C. Harneit A.. (2019). Effects of BDNF Val^66^Met genotype and schizophrenia familial risk on a neural functional network for cognitive control in humans. Neuropsychopharmacology 44, 590–597. doi: 10.1038/s41386-018-0248-9, PMID: 30375508 PMC6333795

[ref146] Sears C. L. (2009). Enterotoxigenic *Bacteroides fragilis*: a rogue among symbiotes. Clin. Microbiol. Rev. 22, 349–369. doi: 10.1128/CMR.00053-08 19366918 PMC2668231

[ref147] Seike M. Goto A. Okano T. Bowman E. D. Schetter A. J. Horikawa I.. (2009). MiR-21 is an EGFR-regulated anti-apoptotic factor in lung cancer in never-smokers. Proc. Natl. Acad. Sci. U.S.A. 106, 12085–12090. doi: 10.1073/pnas.0905234106, PMID: 19597153 PMC2715493

[ref148] Sheikh A. M. Wada Y. Tabassum S. Inagaki S. Mitaki S. Yano S.. (2021). Aggregation of cystatin C changes its inhibitory functions on protease activities and amyloid β fibril formation. Int. J. Mol. Sci. 22:9682. doi: 10.3390/ijms22189682 34575849 PMC8465189

[ref149] Sheinerman K. S. Tsivinsky V. G. Crawford F. Mullan M. J. Abdullah L. Umansky S. R. (2012). Plasma microRNA biomarkers for detection of mild cognitive impairment. Aging 4, 590–605. doi: 10.18632/aging.100486, PMID: 23001356 PMC3492224

[ref150] Shridas P. Tannock L. R. (2019). Role of serum amyloid a in atherosclerosis. Curr. Opin. Lipidol. 30, 320–325. doi: 10.1097/MOL.0000000000000616, PMID: 31135596 PMC7249237

[ref151] Sidorov E. Sanghera D. K. Vanamala J. K. P. (2019). Biomarker for ischemic stroke using metabolome: a clinician perspective. J. Stroke 21, 31–41. doi: 10.5853/jos.2018.03454, PMID: 30732441 PMC6372900

[ref152] Sloley S. S. Main B. S. Winston C. N. Harvey A. C. Kaganovich A. Korthas H. T.. (2021). High-frequency head impact causes chronic synaptic adaptation and long-term cognitive impairment in mice. Nat. Commun. 12:2613. doi: 10.1038/s41467-021-22744-6 33972519 PMC8110563

[ref153] Sorboni S. G. Moghaddam H. S. Jafarzadeh-Esfehani R. Soleimanpour S. (2022). A comprehensive review on the role of the gut microbiome in human neurological disorders. Clin. Microbiol. Rev. 35:e0033820. doi: 10.1128/CMR.00338-20 34985325 PMC8729913

[ref154] Srikanth V. K. Thrift A. G. Saling M. M. Anderson J. F. Dewey H. M. Macdonell R. A.. (2003). Increased risk of cognitive impairment 3 months after mild to moderate first-ever stroke: a community-based prospective study of nonaphasic English-speaking survivors. Stroke 34, 1136–1143. doi: 10.1161/01.STR.0000069161.35736.39, PMID: 12702832

[ref155] Stakos D. A. Stamatelopoulos K. Bampatsias D. Sachse M. Zormpas E. Vlachogiannis N. I.. (2020). The Alzheimer’s disease amyloid-beta hypothesis in cardiovascular aging and disease: JACC focus seminar. J. Am. Coll. Cardiol. 75, 952–967. doi: 10.1016/j.jacc.2019.12.033, PMID: 32130931 PMC7042886

[ref156] Stone T. W. Darlington L. G. (2002). Endogenous kynurenines as targets for drug discovery and development. Nat. Rev. Drug Discov. 1, 609–620. doi: 10.1038/nrd870 12402501

[ref157] Sun J. H. Tan L. Yu J. T. (2014). Post-stroke cognitive impairment: epidemiology, mechanisms and management. Ann. Transl. Med. 2:80. doi: 10.3978/j.issn.2305-5839.2014.08.05 25333055 PMC4200648

[ref158] Szczuko M. Kotlęga D. Palma J. Zembroń-Łacny A. Tylutka A. Gołąb-Janowska M.. (2020). Lipoxins, RevD1 and 9, 13 HODE as the most important derivatives after an early incident of ischemic stroke. Sci. Rep. 10:12849. doi: 10.1038/s41598-020-69831-0 32732956 PMC7393087

[ref159] Sze M. A. Schloss P. D. (2016). Looking for a signal in the noise: revisiting obesity and the microbiome. mBio 7:4. doi: 10.1128/mBio.01018-16 PMC4999546 27555308

[ref160] Tan M. S. Cheah P. L. Chin A. V. Looi L. M. Chang S. W. (2021). A review on omics-based biomarkers discovery for Alzheimer’s disease from the bioinformatics perspectives: statistical approach vs machine learning approach. Comput. Biol. Med. 139:104947. doi: 10.1016/j.compbiomed.2021.104947, PMID: 34678481

[ref161] Tang Y. Xing Y. Zhu Z. He Y. Li F. Yang J.. (2019). The effects of 7-week cognitive training in patients with vascular cognitive impairment, no dementia (the Cog-VACCINE study): a randomized controlled trial. Alzheimers Dement. 15, 605–614. doi: 10.1016/j.jalz.2019.01.009, PMID: 30894299

[ref162] Tein I. De Vivo D. C. Ranucci D. DiMauro S. (1993). Skin fibroblast carnitine uptake in secondary carnitine deficiency disorders. J. Inherit. Metab. Dis. 16, 135–146. doi: 10.1007/BF00711327, PMID: 8387612

[ref163] Teng Z. Dong Y. Zhang D. An J. Lv P. (2017). Cerebral small vessel disease and post-stroke cognitive impairment. Int. J. Neurosci. 127, 824–830. doi: 10.1080/00207454.2016.1261291 27838946

[ref164] Ticinesi A. Nouvenne A. Tana C. Prati B. Meschi T. (2019). Gut microbiota and microbiota-related metabolites as possible biomarkers of cognitive aging. Adv. Exp. Med. Biol. 1178, 129–154. doi: 10.1007/978-3-030-25650-0_8, PMID: 31493226

[ref165] Tsimberidou A. M. Fountzilas E. Bleris L. Kurzrock R. (2022). Transcriptomics and solid tumors: the next frontier in precision cancer medicine. Semin. Cancer Biol. 84, 50–59. doi: 10.1016/j.semcancer.2020.09.007, PMID: 32950605 PMC11927324

[ref166] Ueland P. M. (2011). Choline and betaine in health and disease. J. Inherit. Metab. Dis. 34, 3–15. doi: 10.1007/s10545-010-9088-4, PMID: 20446114

[ref167] Unschuld P. G. Edden R. A. Carass A. Liu X. Shanahan M. Wang X.. (2012). Brain metabolite alterations and cognitive dysfunction in early Huntington's disease. Mov. Disord. 27, 895–902. doi: 10.1002/mds.25010, PMID: 22649062 PMC3383395

[ref168] van den Berg M. M. J. Krauskopf J. Ramaekers J. G. Kleinjans J. C. S. Prickaerts J. Briedé J. J. (2020). Circulating microRNAs as potential biomarkers for psychiatric and neurodegenerative disorders. Prog. Neurobiol. 185:101732. doi: 10.1016/j.pneurobio.2019.101732, PMID: 31816349

[ref169] van Groen T. Puurunen K. Mäki H. M. Sivenius J. Jolkkonen J. (2005). Transformation of diffuse beta-amyloid precursor protein and beta-amyloid deposits to plaques in the thalamus after transient occlusion of the middle cerebral artery in rats. Stroke 36, 1551–1556. doi: 10.1161/01.STR.0000169933.88903.cf, PMID: 15933257

[ref170] Vascellari S. Palmas V. Melis M. Pisanu S. Cusano R. Uva P.. (2020). Gut microbiota and metabolome alterations associated with Parkinson’s disease. mSystems 5:5. doi: 10.1128/mSystems.00561-20 PMC7498685 32934117

[ref171] Verma M. Lizama B. N. Chu C. T. (2022). Excitotoxicity, calcium and mitochondria: a triad in synaptic neurodegeneration. Transl. Neurodegener. 11:3. doi: 10.1186/s40035-021-00278-7 35078537 PMC8788129

[ref172] Veskovic M. Mladenovic D. Milenkovic M. Tosic J. Borozan S. Gopcevic K.. (2019). Betaine modulates oxidative stress, inflammation, apoptosis, autophagy, and Akt/mTOR signaling in methionine-choline deficiency-induced fatty liver disease. Eur. J. Pharmacol. 848, 39–48. doi: 10.1016/j.ejphar.2019.01.043, PMID: 30689995

[ref173] Walgrave H. Balusu S. Snoeck S. Vanden Eynden E. Craessaerts K. Thrupp N.. (2021). Restoring miR-132 expression rescues adult hippocampal neurogenesis and memory deficits in Alzheimer’s disease. Cell Stem Cell 28, 1805–1821.e8. doi: 10.1016/j.stem.2021.05.001, PMID: 34033742

[ref174] Wang Z. Q. Li K. Huang J. Huo T. T. Lv P. Y. (2020). MicroRNA let-7i is a promising serum biomarker for post-stroke cognitive impairment and alleviated OGD-induced cell damage in vitro by regulating Bcl-2. Front. Neurosci. 14:215. doi: 10.3389/fnins.2020.00215, PMID: 32265630 PMC7105869

[ref175] Wang X. Peng Y. Zhou H. Du W. Wang J. Wang J.. (2022). The effects of enriched rehabilitation on cognitive function and serum glutamate levels Post-stroke. Front. Neurol. 13:829090. doi: 10.3389/fneur.2022.829090, PMID: 35370905 PMC8967952

[ref176] Wang Y. Qian P. Y. (2009). Conservative fragments in bacterial 16S rRNA genes and primer design for 16S ribosomal DNA amplicons in metagenomic studies. PLoS One 4:e7401. doi: 10.1371/journal.pone.0007401 19816594 PMC2754607

[ref177] Wang S. Yang H. Zhang J. Zhang B. Liu T. Gan L.. (2016). Efficacy and safety assessment of acupuncture and nimodipine to treat mild cognitive impairment after cerebral infarction: a randomized controlled trial. BMC Complement. Altern. Med. 16:361. doi: 10.1186/s12906-016-1337-0 27623621 PMC5022140

[ref178] Wang H. Zhang M. Li J. Liang J. Yang M. Xia G.. (2022). Gut microbiota is causally associated with poststroke cognitive impairment through lipopolysaccharide and butyrate. J. Neuroinflammation 19:76. doi: 10.1186/s12974-022-02435-9 35379265 PMC8981610

[ref179] Wang R. Zhao N. Li S. Fang J. H. Chen M. X. Yang J.. (2013). MicroRNA-195 suppresses angiogenesis and metastasis of hepatocellular carcinoma by inhibiting the expression of VEGF, VAV2, and CDC42. Hepatology 58, 642–653. doi: 10.1002/hep.26373 23468064

[ref180] Xiayan L. Legido-Quigley C. (2008). Advances in separation science applied to metabonomics. Electrophoresis 29, 3724–3736. doi: 10.1002/elps.200700851 18850642

[ref181] Xie B. Zhou H. Zhang R. Song M. Yu L. Wang L.. (2015). Serum miR-206 and miR-132 as potential circulating biomarkers for mild cognitive impairment. J. Alzheimers Dis. 45, 721–731. doi: 10.3233/JAD-142847, PMID: 25589731

[ref182] Ya D. Zhang Y. Cui Q. Jiang Y. Yang J. Tian N.. (2023). Application of spatial transcriptome technologies to neurological diseases. Front. Cell Dev. Biol. 11:1142923. doi: 10.3389/fcell.2023.1142923 36936681 PMC10020196

[ref183] Yan H. Huang W. Rao J. Yuan J. (2021). miR-21 regulates ischemic neuronal injury via the p53/Bcl-2/Bax signaling pathway. Aging 13, 22242–22255. doi: 10.18632/aging.203530, PMID: 34552038 PMC8507259

[ref184] Yang Z. Wang H. Edwards D. Ding C. Yan L. Brayne C.. (2020). Association of blood lipids, atherosclerosis and statin use with dementia and cognitive impairment after stroke: a systematic review and meta-analysis. Ageing Res. Rev. 57:100962. doi: 10.1016/j.arr.2019.100962, PMID: 31505259

[ref185] Yang S. Zhan X. He M. Wang J. Qiu X. (2020). miR-135b levels in the peripheral blood serve as a marker associated with acute ischemic stroke. Exp. Ther. Med. 19, 3551–3558. doi: 10.3892/etm.2020.8628, PMID: 32346417 PMC7185079

[ref186] Ye J. Xu M. Tian X. Cai S. Zeng S. (2019). Research advances in the detection of miRNA. J. Pharm. Anal. 9, 217–226. doi: 10.1016/j.jpha.2019.05.004, PMID: 31452959 PMC6702429

[ref187] Yoshida T. Ishikawa M. Niitsu T. Nakazato M. Watanabe H. Shiraishi T.. (2012). Decreased serum levels of mature brain-derived neurotrophic factor (BDNF), but not its precursor proBDNF, in patients with major depressive disorder. PLoS One 7:e42676. doi: 10.1371/journal.pone.0042676 22880079 PMC3411809

[ref188] Yuan M. Guo Y. S. Zhang X. X. Gao Z. K. Shen X. Y. Han Y.. (2022). Diagnostic performance of miR-21, miR-124, miR-132, and miR-200b serums in post-stroke cognitive impairment patients. Folia Neuropathol. 60, 228–236. doi: 10.5114/fn.2022.118187, PMID: 35950475

[ref189] Zeng Q. Huang Z. Wei L. Fang J. Lin K. (2019). Correlations of serum cystatin C level and gene polymorphism with vascular cognitive impairment after acute cerebral infarction. Neurol. Sci. 40, 1049–1054. doi: 10.1007/s10072-019-03777-8, PMID: 30805744

[ref190] Zhai Y. Zhu Z. Li H. Zhao C. Huang Y. Wang P. (2020). miR-195 and miR-497 in acute stroke and their correlations with post-stroke cognitive impairment. Int. J. Clin. Exp. Pathol. 13, 3092–3099, PMID: 33425109 PMC7791374

[ref191] Zhang Y. Feng Y. Zuo J. Shi J. Zhang S. Yang Y.. (2021). Elevated serum amyloid a is associated with cognitive impairment in ischemic stroke patients. Front. Neurol. 12:789204. doi: 10.3389/fneur.2021.789204 35111127 PMC8801533

[ref192] Zhang J. Li S. Li L. Li M. Guo C. Yao J.. (2015). Exosome and exosomal microRNA: trafficking, sorting, and function. Genomics Proteomics Bioinformatics 13, 17–24. doi: 10.1016/j.gpb.2015.02.001, PMID: 25724326 PMC4411500

[ref193] Zhang A. H. Sun H. Wang X. J. (2013). Recent advances in metabolomics in neurological disease, and future perspectives. Anal. Bioanal. Chem. 405, 8143–8150. doi: 10.1007/s00216-013-7061-4, PMID: 23715678

[ref194] Zhang X. Yuan M. Yang S. Chen X. Wu J. Wen M.. (2021). Enriched environment improves post-stroke cognitive impairment and inhibits neuroinflammation and oxidative stress by activating Nrf2-ARE pathway. Int. J. Neurosci. 131, 641–649. doi: 10.1080/00207454.2020.1797722, PMID: 32677581

[ref195] Zhang J. Zhang Y. Li J. Xing S. Li C. Li Y.. (2012). Autophagosomes accumulation is associated with β-amyloid deposits and secondary damage in the thalamus after focal cortical infarction in hypertensive rats. J. Neurochem. 120, 564–573. doi: 10.1111/j.1471-4159.2011.07496.x, PMID: 21950964

[ref196] Zhang R. Zhang H. Zhang Z. Wang T. Niu J. Cui D.. (2012). Neuroprotective effects of pre-treatment with l-carnitine and acetyl-L-carnitine on ischemic injury *in vivo* and *in vitro*. Int. J. Mol. Sci. 13, 2078–2090. doi: 10.3390/ijms13022078, PMID: 22408439 PMC3292008

[ref197] Zhao J. Liu S. Yan J. Zhu X. (2021). The impact of gut microbiota on post-stroke management. Front. Cell. Infect. Microbiol. 11:724376. doi: 10.3389/fcimb.2021.724376, PMID: 34712621 PMC8546011

[ref198] Zhong C. Lu Z. Che B. Qian S. Zheng X. Wang A.. (2021). Choline pathway nutrients and metabolites and cognitive impairment after acute ischemic stroke. Stroke 52, 887–895. doi: 10.1161/STROKEAHA.120.031903 33467878

[ref199] Zhou Q. Liu J. Quan J. Liu W. Tan H. Li W. (2018). MicroRNAs as potential biomarkers for the diagnosis of glioma: a systematic review and meta-analysis. Cancer Sci. 109, 2651–2659. doi: 10.1111/cas.13714, PMID: 29949235 PMC6125451

[ref200] Zhu C. Li G. Lv Z. Li J. Wang X. Kang J.. (2020). Association of plasma trimethylamine-N-oxide levels with post-stroke cognitive impairment: a 1-year longitudinal study. Neurol. Sci. 41, 57–63. doi: 10.1007/s10072-019-04040-w, PMID: 31420758

