# Supplementary material for: Post-stroke cognitive impairment: exploring molecular mechanisms and omics biomarkers for early identification and intervention
Source: Front Mol Neurosci. 2024 May 23;17:1375973. doi: 10.3389/fnmol.2024.1375973 (PMC11153683; doi:10.3389/fnmol.2024.1375973)
Supplement: Supplementary file 1 [file Table_1.DOCX]

Supplementary Material

# Table S1. Metabolomics key terms for search of electronic database

| **Key terms for search of PubMed** | |
| --- | --- |
| #1 | "magnetic Resonance Spectroscopy" [Mesh] OR "Chromatography" [Mesh] OR "Mass spectrometry" [Mesh] OR "liquid chromatograph*" OR "gas chromatograph*" OR "nuclear magnetic resonance" OR chromatograph* |
| #2 | "metabolome" [Mesh] OR "metabolomics" [Mesh] OR metabolo* OR metabonom* OR "metabolite network*" OR "metabolite profile*" OR lipidom* OR biomarker*[Mesh] OR "biological marker*" OR "biochemical marker*" OR "amino acid turnover" OR "amino acid metabolism" OR metabolite |
| #3 | 1 OR 2 |
| #4 | stroke OR stroke [Mesh] OR "cerebral infarct" OR "brain infarct" OR "cerebral hemorrhage" OR "cerebral ischemia" OR "cerebral hematoma" OR "brain hemorrhage" |
| #5 | dementia OR dementia [Mesh] OR "cognitive decline" OR "cognitive impairment" OR cognition disorder OR cognition disorders [Mesh] |
| #6 | 3 AND 4 AND 5 |
| **Key terms for search of Cochrane Library** | |
| #1 | MeSH descriptor: [Stroke] explode all trees |
| #2 | MeSH descriptor: [Cerebral Infarction] explode all trees |
| #3 | MeSH descriptor: [Brain Ischemia] explode all trees |
| #4 | MeSH descriptor: [Brain Infarction] explode all trees |
| #5 | MeSH descriptor: [Cerebral Hemorrhage] explode all trees |
| #6 | MeSH descriptor: [Hemorrhagic Stroke] explode all trees |
| #7 | MeSH descriptor: [Ischemic Stroke] explode all trees |
| #8 | MeSH descriptor: [Cerebrovascular Disorders] explode all trees |
| #9 | (stroke or poststroke or post‐stroke or cerebrovasc* or (cerebr* near/3 vasc*) or CVA* or apoplectic or apoplex* or (transient near/3 isch?emic near/3 attack) or tia* or SAH or AVM or ESUS or ICH or (cerebral small vessel near/3 disease*)):ti,ab,kw |
| #10 | #1 OR #2 OR #3 OR #4 OR #5 OR #6 OR #7 OR #8 ORv#9 |
| #11 | MeSH descriptor: [Dementia] explode all trees |
| #12 | MeSH descriptor: [Cognition Disorders] explode all trees |
| #13 | MeSH descriptor: [Cognitive Dysfunction] explode all trees |
| #14 | (dementia or dement* or cogniti*):ti,ab,kw |
| #15 | #11 OR #12 OR #13 OR #14 |
| #16 | #10 AND #15 |
| #17 | MeSH descriptor: [Metabolomics] explode all trees |
| #18 | MeSH descriptor: [Metabolome] explode all trees |
| #19 | MeSH descriptor: [Magnetic Resonance Spectroscopy] explode all trees |
| #20 | (metabolomics OR metabolome OR metabolo* OR metabolite OR metabolite network* OR metabolite profil* OR lipidom* OR biomarker*):ti,ab,kw |
| #21 | (magnetic Resonance Spectroscopy OR Chromatography OR Mass spectrometry OR liquid chromatograph* OR gas chromatograph* OR nuclear magnetic resonance OR chromatograph*):ti,ab,kw |
| #22 | #17 OR #18 OR 19 OR #20 OR #21 |
| #23 | #16 AND #22 |
| **Key terms for search of Embase** | |
| #1 | ('magnetic Resonance Spectroscopy' OR 'Chromatography’ OR 'Mass spectrometry' OR 'liquid chromatograph*' OR 'gas chromatograph*' OR 'nuclear magnetic resonance' OR 'chromatograph*'):ab,ti |
| #2 | ('metabolome' OR 'metabolomics' OR 'metabolo*':ab,ti OR 'metabonom*':ab,ti OR 'metabolite network*':ab,ti OR 'metabolite profil*':ab,ti OR 'lipidom*':ab,ti OR 'biomarker*':ab,ti OR 'biological marker*':ab,ti OR 'biochemical marker*':ab,ti OR 'amino acid turnover':ab,ti OR 'amino acid metabolism':ab,ti OR 'metabolite'):ti,ab |
| #3 | #1 OR #2 |
| #4 | 'stroke'/exp OR ('cerebral infarct' OR 'brain infarct' OR 'cerebral hemorrhage' OR 'cerebral ischemia' OR 'cerebral hematoma' OR 'brain hemorrhage'):ab,ti |
| #5 | 'dementia'/exp OR ('cognition disorders' OR 'cognitive decline' OR 'cognitive impairment'):ab,ti |
| #6 | #3 AND #4 AND #5 |
| **Key terms for search of Web of Science** | |
| #1 | TS=("magnetic Resonance Spectroscopy" OR Chromatography OR "Mass spectrometry" OR "liquid chromatograph*" OR "gas chromatograph*" OR "nuclear magnetic resonance" OR chromatograph*) |
| #2 | TS=(metabolome OR metabolomics OR metabolo* OR metabonom* OR "metabolite network*" OR "metabolite profile*" OR lipidom* OR biomarker* OR "biological marker*" OR "biochemical marker*" OR "amino acid turnover" OR "amino acid metabolism" OR metabolite) |
| #3 | #1 OR #2 |
| #4 | TS=(stroke OR "cerebral infarct" OR "brain infarct" OR "cerebral hemorrhage" OR "cerebral ischemia" OR "cerebral hematoma" OR "brain hemorrhage") |
| #5 | TS=(dementia OR "cognitive decline" OR "cognitive impairment" OR “cognition disorder”) |
| #6 | #3 AND #4 AND #5 |

# Table S2. Gut Microbiomics key terms for search of electronic database.

| **Key terms for search of PubMed** | |
| --- | --- |
| #1 | stroke OR stroke [Mesh] OR "cerebral infarct" OR "brain infarct" OR "cerebral hemorrhage" OR "cerebral ischemia" OR "cerebral hematoma" OR "brain hemorrhage" |
| #2 | dementia OR dementia [Mesh] OR "cognitive decline" OR "cognitive impairment" OR cognition disorder OR cognition disorders [Mesh] |
| #3 | 1 AND 2 |
| #4 | gut OR microbiota OR microbiome |
| #5 | 3 AND 4 |
| **Key terms for search of Cochrane Library** | |
| #1 | MeSH descriptor: [Stroke] explode all trees |
| #2 | MeSH descriptor: [Cerebral Infarction] explode all trees |
| #3 | MeSH descriptor: [Brain Ischemia] explode all trees |
| #4 | MeSH descriptor: [Brain Infarction] explode all trees |
| #5 | MeSH descriptor: [Cerebral Hemorrhage] explode all trees |
| #6 | MeSH descriptor: [Hemorrhagic Stroke] explode all trees |
| #7 | MeSH descriptor: [Ischemic Stroke] explode all trees |
| #8 | MeSH descriptor: [Cerebrovascular Disorders] explode all trees |
| #9 | (stroke or poststroke or post‐stroke or cerebrovasc* or (cerebr* near/3 vasc*) or CVA* or apoplectic or apoplex* or (transient near/3 isch?emic near/3 attack) or tia* or SAH or AVM or ESUS or ICH or (cerebral small vessel near/3 disease*)):ti,ab,kw |
| #10 | #1 OR #2 OR #3 OR #4 OR #5 OR #6 OR #7 OR #8 ORv#9 |
| #11 | MeSH descriptor: [Dementia] explode all trees |
| #12 | MeSH descriptor: [Cognition Disorders] explode all trees |
| #13 | MeSH descriptor: [Cognitive Dysfunction] explode all trees |
| #14 | (dementia or dement* or cogniti*):ti,ab,kw |
| #15 | #11 OR #12 OR #13 OR #14 |
| #16 | #10 AND #15 |
| #17 | MeSH descriptor: [Gastrointestinal Microbiome] explode all trees |
| #18 | MeSH descriptor: [Microbiota] explode all trees |
| #19 | (microbiota OR Gastrointestinal Microbiome OR microbio* OR gut microbiome):ti,ab,kw |
| #20 | #17 OR #18 OR #19 |
| #21 | #16 AND #20 |
| **Key terms for search of Embase** | |
| #1 | 'microbiome'/exp OR 'microbiomics'/exp OR 'microbiota'/exp OR ('gastrointestinal microbiome' OR 'gut microbiome'):ab,ti |
| #2 | 'stroke'/exp OR ('cerebral infarct' OR 'brain infarct' OR 'cerebral hemorrhage' OR 'cerebral ischemia' OR 'cerebral hematoma' OR 'brain hemorrhage'):ab,ti |
| #3 | 'dementia'/exp OR ('cognition disorders' OR 'cognitive decline' OR 'cognitive impairment'):ab,ti |
| #4 | #1 AND #2 AND #3 |
| **Key terms for search of Web of Science** | |
| #1 | TS=(stroke OR "cerebral infarct" OR "brain infarct" OR "cerebral hemorrhage" OR "cerebral ischemia" OR "cerebral hematoma" OR "brain hemorrhage") |
| #2 | TS=(dementia OR "cognitive decline" OR "cognitive impairment" OR “cognition disorder”) |
| #3 | TS=(microbiome OR microbiomics OR microbiota OR Gastrointestinal Microbiome OR gut microbiome) |
| #4 | #1 AND #2 AND #3 |

# Table S3. Genomics key terms for search of electronic database.

| **Key terms for search of PubMed** | |
| --- | --- |
| #1 | stroke OR stroke [Mesh] OR "cerebral infarct" OR "brain infarct" OR "cerebral hemorrhage" OR "cerebral ischemia" OR "cerebral hematoma" OR "brain hemorrhage" |
| #2 | dementia OR dementia [Mesh] OR "cognitive decline" OR "cognitive impairment" OR cognition disorder OR cognition disorders [Mesh] |
| #3 | 1 AND 2 |
| #4 | DNA OR gene OR genom* |
| #5 | 3 AND 4 |
| **Key terms for search of Cochrane Library** | |
| #1 | MeSH descriptor: [Stroke] explode all trees |
| #2 | MeSH descriptor: [Cerebral Infarction] explode all trees |
| #3 | MeSH descriptor: [Brain Ischemia] explode all trees |
| #4 | MeSH descriptor: [Brain Infarction] explode all trees |
| #5 | MeSH descriptor: [Cerebral Hemorrhage] explode all trees |
| #6 | MeSH descriptor: [Hemorrhagic Stroke] explode all trees |
| #7 | MeSH descriptor: [Ischemic Stroke] explode all trees |
| #8 | MeSH descriptor: [Cerebrovascular Disorders] explode all trees |
| #9 | (stroke or poststroke or post‐stroke or cerebrovasc* or (cerebr* near/3 vasc*) or CVA* or apoplectic or apoplex* or (transient near/3 isch?emic near/3 attack) or tia* or SAH or AVM or ESUS or ICH or (cerebral small vessel near/3 disease*)):ti,ab,kw |
| #10 | #1 OR #2 OR #3 OR #4 OR #5 OR #6 OR #7 OR #8 ORv#9 |
| #11 | MeSH descriptor: [Dementia] explode all trees |
| #12 | MeSH descriptor: [Cognition Disorders] explode all trees |
| #13 | MeSH descriptor: [Cognitive Dysfunction] explode all trees |
| #14 | (dementia or dement* or cogniti*):ti,ab,kw |
| #15 | #11 OR #12 OR #13 OR #14 |
| #16 | #10 AND #15 |
| #17 | MeSH descriptor: [DNA] explode all trees |
| #18 | MeSH descriptor: [Genes] explode all trees |
| #19 | MeSH descriptor: [Genome] explode all trees |
| #20 | MeSH descriptor: [Genomics] explode all trees |
| #21 | (genome OR genomics OR gene OR DNA):ti,ab,kw |
| #22 | #17 OR #18 OR #19 OR #20 OR #21 |
| #23 | #16 AND #22 |
| **Key terms for search of Embase** | |
| #1 | 'genome'/exp OR 'genomics'/exp OR 'gene'/exp OR 'dna'/exp |
| #2 | 'stroke'/exp OR ('cerebral infarct' OR 'brain infarct' OR 'cerebral hemorrhage' OR 'cerebral ischemia' OR 'cerebral hematoma' OR 'brain hemorrhage'):ab,ti |
| #3 | 'dementia'/exp OR ('cognition disorders' OR 'cognitive decline' OR 'cognitive impairment'):ab,ti |
| #4 | #1 AND #2 AND #3 |
| **Key terms for search of Web of Science** | |
| #1 | TS=(stroke OR "cerebral infarct" OR "brain infarct" OR "cerebral hemorrhage" OR "cerebral ischemia" OR "cerebral hematoma" OR "brain hemorrhage") |
| #2 | TS=(dementia OR "cognitive decline" OR "cognitive impairment" OR “cognition disorder”) |
| #3 | TS=(genome OR genomics OR gene OR DNA) |
| #4 | #1 AND #2 AND #3 |

# Table S4. Transcriptomics key terms for search of electronic database.

| **Key terms for search of PubMed** | |
| --- | --- |
| #1 | stroke OR stroke [Mesh] OR "cerebral infarct" OR "brain infarct" OR "cerebral hemorrhage" OR "cerebral ischemia" OR "cerebral hematoma" OR "brain hemorrhage" |
| #2 | dementia OR dementia [Mesh] OR "cognitive decline" OR "cognitive impairment" OR cognition disorder OR cognition disorders [Mesh] |
| #3 | 1 AND 2 |
| #4 | transcription OR RNA OR gene express OR transcriptome* |
| #5 | 3 AND 4 |
| **Key terms for search of Cochrane Library** | |
| #1 | MeSH descriptor: [Stroke] explode all trees |
| #2 | MeSH descriptor: [Cerebral Infarction] explode all trees |
| #3 | MeSH descriptor: [Brain Ischemia] explode all trees |
| #4 | MeSH descriptor: [Brain Infarction] explode all trees |
| #5 | MeSH descriptor: [Cerebral Hemorrhage] explode all trees |
| #6 | MeSH descriptor: [Hemorrhagic Stroke] explode all trees |
| #7 | MeSH descriptor: [Ischemic Stroke] explode all trees |
| #8 | MeSH descriptor: [Cerebrovascular Disorders] explode all trees |
| #9 | (stroke or poststroke or post‐stroke or cerebrovasc* or (cerebr* near/3 vasc*) or CVA* or apoplectic or apoplex* or (transient near/3 isch?emic near/3 attack) or tia* or SAH or AVM or ESUS or ICH or (cerebral small vessel near/3 disease*)):ti,ab,kw |
| #10 | #1 OR #2 OR #3 OR #4 OR #5 OR #6 OR #7 OR #8 ORv#9 |
| #11 | MeSH descriptor: [Dementia] explode all trees |
| #12 | MeSH descriptor: [Cognition Disorders] explode all trees |
| #13 | MeSH descriptor: [Cognitive Dysfunction] explode all trees |
| #14 | (dementia or dement* or cogniti*):ti,ab,kw |
| #15 | #11 OR #12 OR #13 OR #14 |
| #16 | #10 AND #15 |
| #17 | MeSH descriptor: [Transcription, Genetic] explode all trees |
| #18 | MeSH descriptor: [Transcriptome] explode all trees |
| #19 | MeSH descriptor: [Gene Expression Profiling] explode all trees |
| #20 | MeSH descriptor: [RNA-Seq] explode all trees |
| #21 | (transcription OR transcriptome OR transcriptom* OR gene express* OR RNA):ti,ab,kw |
| #22 | #17 OR #18 OR #19 OR #20 OR #21 |
| #23 | #16 AND #22 |
| **Key terms for search of Embase** | |
| #1 | 'transcription'/exp OR 'transcriptome'/exp OR 'transcriptomics'/exp OR ('gene express*' OR 'RNA'):ab,ti |
| #2 | 'stroke'/exp OR ('cerebral infarct' OR 'brain infarct' OR 'cerebral hemorrhage' OR 'cerebral ischemia' OR 'cerebral hematoma' OR 'brain hemorrhage'):ab,ti |
| #3 | 'dementia'/exp OR ('cognition disorders' OR 'cognitive decline' OR 'cognitive impairment'):ab,ti |
| #4 | #1 AND #2 AND #3 |
| **Key terms for search of Web of Science** | |
| #1 | TS=(stroke OR "cerebral infarct" OR "brain infarct" OR "cerebral hemorrhage" OR "cerebral ischemia" OR "cerebral hematoma" OR "brain hemorrhage") |
| #2 | TS=(dementia OR "cognitive decline" OR "cognitive impairment" OR “cognition disorder”) |
| #3 | TS=(transcription OR transcriptome OR transcriptomics OR gene express* OR RNA) |
| #4 | #1 AND #2 AND #3 |

# Table S5. Proteomics key terms for search of electronic database.

| **Key terms for search of PubMed** | |
| --- | --- |
| #1 | stroke OR stroke [Mesh] OR "cerebral infarct" OR "brain infarct" OR "cerebral hemorrhage" OR "cerebral ischemia" OR "cerebral hematoma" OR "brain hemorrhage" |
| #2 | dementia OR dementia [Mesh] OR "cognitive decline" OR "cognitive impairment" OR cognition disorder OR cognition disorders [Mesh] |
| #3 | 1 AND 2 |
| #4 | proteome OR proteome [Mesh]OR proteom* OR "proteome profile*" |
| #5 | 3 AND 4 |
| **Key terms for search of Cochrane Library** | |
| #1 | MeSH descriptor: [Stroke] explode all trees |
| #2 | MeSH descriptor: [Cerebral Infarction] explode all trees |
| #3 | MeSH descriptor: [Brain Ischemia] explode all trees |
| #4 | MeSH descriptor: [Brain Infarction] explode all trees |
| #5 | MeSH descriptor: [Cerebral Hemorrhage] explode all trees |
| #6 | MeSH descriptor: [Hemorrhagic Stroke] explode all trees |
| #7 | MeSH descriptor: [Ischemic Stroke] explode all trees |
| #8 | MeSH descriptor: [Cerebrovascular Disorders] explode all trees |
| #9 | (stroke or poststroke or post‐stroke or cerebrovasc* or (cerebr* near/3 vasc*) or CVA* or apoplectic or apoplex* or (transient near/3 isch?emic near/3 attack) or tia* or SAH or AVM or ESUS or ICH or (cerebral small vessel near/3 disease*)):ti,ab,kw |
| #10 | #1 OR #2 OR #3 OR #4 OR #5 OR #6 OR #7 OR #8 ORv#9 |
| #11 | MeSH descriptor: [Dementia] explode all trees |
| #12 | MeSH descriptor: [Cognition Disorders] explode all trees |
| #13 | MeSH descriptor: [Cognitive Dysfunction] explode all trees |
| #14 | (dementia or dement* or cogniti*):ti,ab,kw |
| #15 | #11 OR #12 OR #13 OR #14 |
| #16 | #10 AND #15 |
| #17 | MeSH descriptor: [Proteomics] explode all trees |
| #18 | MeSH descriptor: [Proteome] explode all trees |
| #19 | (proteome OR proteomics OR proteom* OR proteome profil*):ti,ab,kw |
| #20 | #17 OR #18 OR #19 |
| #21 | #16 AND #20 |
| **Key terms for search of Embase** | |
| #1 | 'proteome'/exp OR 'proteomics'/exp OR ('proteom*' OR 'proteome profil*'):ab,ti |
| #2 | 'stroke'/exp OR ('cerebral infarct' OR 'brain infarct' OR 'cerebral hemorrhage' OR 'cerebral ischemia' OR 'cerebral hematoma' OR 'brain hemorrhage'):ab,ti |
| #3 | 'dementia'/exp OR ('cognition disorders' OR 'cognitive decline' OR 'cognitive impairment'):ab,ti |
| #4 | #1 AND #2 AND #3 |
| **Key terms for search of Web of Science** | |
| #1 | TS=(stroke OR "cerebral infarct" OR "brain infarct" OR "cerebral hemorrhage" OR "cerebral ischemia" OR "cerebral hematoma" OR "brain hemorrhage") |
| #2 | TS=(dementia OR "cognitive decline" OR "cognitive impairment" OR “cognition disorder”) |
| #3 | TS=(proteome OR proteomics OR proteome* OR "proteome profile*") |
| #4 | #1 AND #2 AND #3 |

# Table S6. Search results of metabolomics for each database.

| **Databases** | **URL** | **Citations** |
| --- | --- | --- |
| **Literature databases:** |  |  |
| PubMed | http://www.ncbi.nlm.nih.gov/pubmed | 1,267 |
| Cochrane Library | http://onlinelibrary.wiley.com/cochranelibrary/search | 1,640 |
| Embase | http://www.embase.com | 1,201 |
| Web of Science | http://www.webofknowledge.com | 1,907 |
| **Total (literature databases)** | | **6,015** |
|  |  |  |
| **Metabolomics databases:** |  |  |
| HMDB | http://www.hmdb.ca | 202 |
| MetaboLights | http://www.ebi.ac.uk/metabolights | 286 |
| MetabolomeXchange | http://www.metabolomexchange.org/site | 1 |
| Omics Discovery Index | http://www.omicsdi.org/home | 178 |
| **Total (metabolomics databases)** | | **667** |

# Table S7. Search results of gut microbiomics for each database.

| **Databases** | **URL** | **Citations** |
| --- | --- | --- |
| **Literature databases:** |  |  |
| PubMed | http://www.ncbi.nlm.nih.gov/pubmed | 110 |
| Cochrane Library | http://onlinelibrary.wiley.com/cochranelibrary/search | 30 |
| Embase | http://www.embase.com | 159 |
| Web of Science | http://www.webofknowledge.com | 101 |
| **Total (literature databases)** | | **400** |
|  |  |  |
| **Gut microbiomics databases** |  |  |
| Sequence Read Archive | www.ncbi.nlm.nih.gov/sra | 46 |
| European Nucleotide Archive | www.ebi.ac.uk/ena | 4 |
| **Total** | | **50** |

# Table S8. Search results of genomics for each database.

| **Databases** | **URL** | **Citations** |
| --- | --- | --- |
| **Literature databases:** |  |  |
| PubMed | www.ncbi.nlm.nih.gov/pubmed | 2,657 |
| Cochrane Library | www.onlinelibrary.wiley.com/cochranelibrary | 138 |
| Embase | www.embase.com | 1,059 |
| Web of Science | www.webofknowledge.com | 2,969 |
| **Total (literature databases)** | | **6,823** |
|  |  |  |
| **Genomics databases:** |  |  |
| OMIM | www.omim.org | 717 |
| CNGB | www.db.cngb.org | 4 |
| GIGA | http://gigadb.org/ | 0 |
| **Total** | | **721** |

# Table S9. Search results of transcriptomics for each database.

| **Databases** | **URL** | **Citations** |
| --- | --- | --- |
| **Literature databases:** |  |  |
| PubMed | http://www.ncbi.nlm.nih.gov/pubmed | 1,106 |
| Cochrane Library | http://onlinelibrary.wiley.com/cochranelibrary/search | 108 |
| Embase | http://www.embase.com | 413 |
| Web of Science | http://www.webofknowledge.com | 1,623 |
| **Total (literature databases)** | | **3,250** |
|  |  |  |
| **Transcriptomics databases:** |  |  |
| NCBI | www.ncbi.nlm.nih.gov/gds/ | 52 |
| EBI | www.ebi.ac.uk/biostudies/arrayexpress | 624 |
| Ensembl | www. asia.ensembl.org | 157 |
| **Total** | | **833** |

# Table S10. Search results of proteomics for each database.

| **Databases** | **URL** | **Citations** |
| --- | --- | --- |
| **Literature databases:** |  |  |
| PubMed | http://www.ncbi.nlm.nih.gov/pubmed | 139 |
| Cochrane Library | http://onlinelibrary.wiley.com/cochranelibrary/search | 10 |
| Embase | http://www.embase.com | 130 |
| Web of Science | http://www.webofknowledge.com | 71 |
| **Total (literature databases)** | | **350** |
|  |  |  |
| **Proteomics databases:** |  |  |
| Uniprot | www.uniprot.org | 1 |
| InterPro | www.ebi.ac.uk/interpro | 0 |
| CDD | www.ncbi.nlm.nih.gov/Structure/cdd/cdd.shtml | 2 |
| KEGG | www.genome.jp/kegg | 0 |
| **Total** | | **3** |
